# Selective Halogen-Lithium Exchange of 1,2-Dihaloarenes for Successive [2+4] Cycloadditions of Arynes and Isobenzofurans

**DOI:** 10.3390/molecules201019449

**Published:** 2015-10-23

**Authors:** Shohei Eda, Toshiyuki Hamura

**Affiliations:** Department of Applied Chemistry for Environment, School of Science and Technology, Kwansei Gakuin University, 2-1 Gakuen, Sanda, Hyogo 669-1337, Japan; E-Mail: shoheie816@gmail.com

**Keywords:** aryne, isobenzofuran, [2+4] cycloaddition, 1,2-dihaloarenes, polyacene, halogen-lithium exchange

## Abstract

Successive [2+4] cycloadditions of arynes and isobenzofurans by site-selective halogen-lithium exchange of 1,2-dihaloarenes were developed, allowing the rapid construction of polycyclic compounds which serve as a useful synthetic intermediates for the preparation of various polyacene derivatives.

## 1. Introduction

**Scheme 1:**
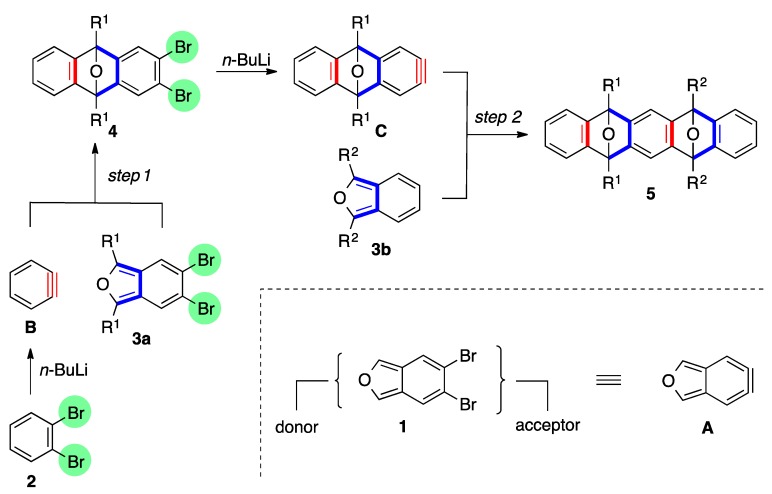
Successive [2+4] cycloadditions of arynes and isobenzofurans.

We previously reported dual annulations of dibromoisobenzofuran **1**, a formal equivalent of didehydroisobenzofuran **A**, via [2+4] cycloadditions of aryne [1,2,3,4,5,6,7,8,9] and isobenzofuran [10,11,12,13,14,15,16,17,18,19,20,21,22,23] (Scheme 1). Selective bromine–lithium exchange from the starting two dibromides **2** and **3** enables the tandem generation of arynes and dual cycloadditions with two different arynophiles (step 1 and step 2). Importantly, successive process can be performed in one-pot by sequential addition of the arynophiles, affording various functionalized polycyclic aromatic compounds [24,25,26].

**Scheme 2:**
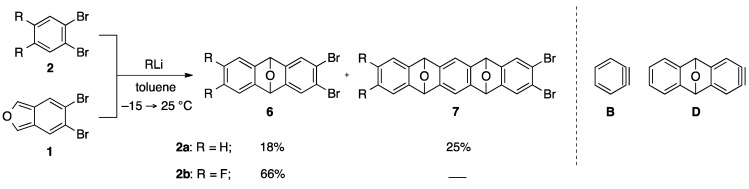
Previous study on the [2+4] cycloaddition of benzyne and dibromoisobenzofuran.

This sequential cycloaddition, however, has a limitation in that the introduction of electron withdrawing groups on the benzene ring in aryne precursor (e.g., **2b**) is required to restrict the competitive formation of the dual cycloadduct (Scheme 2). In fact, treatment of dibromobenzene **2a** with *n*-BuLi in the presence of dibromoisobenzofuran **1** gave cycloadduct **6a** in 18% yield, accompanied by a sizable amount of bis-cycloadduct **7a** (25%). This result indicates that in addition to the generation of benzyne **B**, similar reactivity of two dibromides **2a** and **6a** with *n*-BuLi caused the competitive generation of aryne **D** from the initially formed cycloadduct **6a**. In this case, excess amounts of the starting material **2a** (5.0 equiv.) improved the yields of the mono-cycloadduct **6a** (42%) by selective generation of benzyne **B**. However, it is not an essential solution, since existing of the large amount of the starting material **2a** disturbed the second [2+4] cycloaddition with **6a** in a one-pot process.

**Scheme 3:**
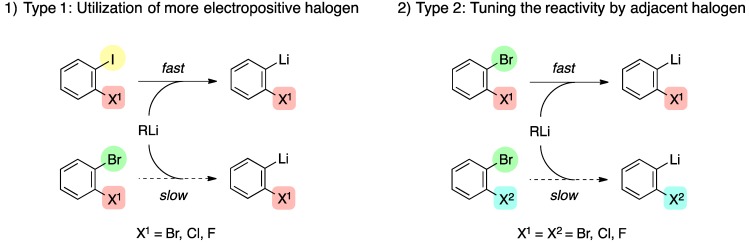
Two strategies for selective halogen-lithium exchange of 1,2-dihaloarenes.

To expand the synthetic utility of this successive processes, we reexamined [2+4] cycloadditions of aryne and isobenzofuran including the parent benzyne species **B** as an initial cycloaddition (*vide supra*). The key to achieve this sequential process is search for a suitable aryne precursor to enable the selective halogen-lithium exchange [27,28,29,30]. Along these lines, we select 1,2-dihaloarenes as an aryne precursor and expect that controlling the reactivity of the halogen would be possible by taking advantage of the following two features: (1) utilization of the more electropositive halogen (type 1); or (2) tuning the reactivity of halogen by the adjacent halogen (type 2) as shown in Scheme 3. The naive idea of the second strategy is that the strong electron-withdrawing ability of the adjacent halogen might reinforce the electrophilicity of the halogen atom, thus facilitating the halogen-lithium exchange. Importantly, these two factors would allow for the site-selective halogen-lithium exchange among three halides, *i.e.*, dihaloarene, dihaloisobenzofuran, and dihalocycloadduct (Scheme 1), which leads to the tandem generation of arynes and multiple cycloadditions with two or three different arynophiles. We report herein the positive resolution of this scenario [31,32].

## 2. Results and Discussion

Table 1 shows initial model reaction for selective generation of benzyne species **B**. Upon treatment of 1-bromo-2-iodobenzene (**8a**) with 1.2 equiv. of *n*-BuLi in the presence of 1.0 equiv. of 5,6-dibromoisobenzofuran (**1**) in toluene at −78 °C, iodine–lithium exchange of **8a** occurred cleanly. The aryllithium intermediate, thus formed, underwent 1,2-elimination of LiBr to generate benzyne **B**, which was trapped with **1** to give mono-cycloadduct **6a** in 60% yield (entry 1). It is clear that the formation of the bis-cycloadduct **7a** via the bromine-lithium exchange of **6a** was not fully but mainly suppressed (9%) in comparison with the corresponding reaction of dibromide **2a** used as a benzyne source. Same reaction at higher reaction temperature (−15→25 °C) gave a better yield of the desired product **6a** (78%), and the bis-cycloadduct **7a** was obtained only in 1% yield (entry 2). Using 1-chloro-2-iodobenzene (**8b**) as a benzyne precursor again proved feasible with *n*-BuLi (toluene, −15→25 °C), affording **6a** in 62% yield (entry 4). Moreover, the corresponding reaction of iodide **8c** having a fluorine atom at 2-position as a leaving group gave moderate yield of **6a** (entries 5–6). These results indicate that halogen-lithium exchange selectively occurred at the more electropositive iodine atom in iodo-halides **8a**–**8c** (Type 1 in Scheme 3), smoothly generating (2-halo)phenyllithiums, respectively, whereas the dibromoisobenzofuran **1** and the dibromocycloadduct **6a** almost untouched under these conditions [33,34,35,36]. As for the moderate yield of the cycloadduct **6a** in the reaction of the dihalides **8b** and **8c**, the lower leaving ability of halogen (Cl and F) in comparison with bromine in aryllithium species would affect the elimination of lithium halide and subsequent generation of benzyne **B** [37]. Based on these reaction outcomes, it is safe to say that use of 1,2-dihaloarenes **8a**–**8c** possessing a more electropositive iodine atom is favored as a benzyne precursor over the bromide **2a** in terms of selectivity and yield.

**Table 1:** Initial model study. 
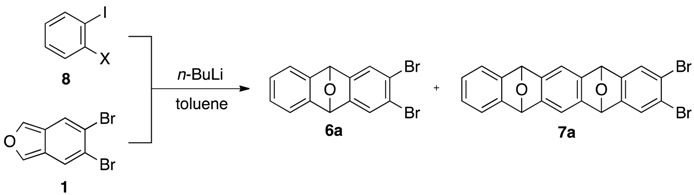

Entry	X	Temp. (°C)	Yield of 6a (%)	Yield of 7a (%) ^1^
1	**8a**: Br	–78	60	9
2	**8a**: Br	–15→25	78	1
3	**8b**: Cl	–78	51	9
4	**8b**: Cl	–15→25	62	9
5	**8c**: F	–78	41	4
6	**8c**: F	–15→25	44	11

^1^ The cycloadduct **7a** was obtained as a mixture of disastereomers (ds: 44/56~58/42).

**Scheme 4:**
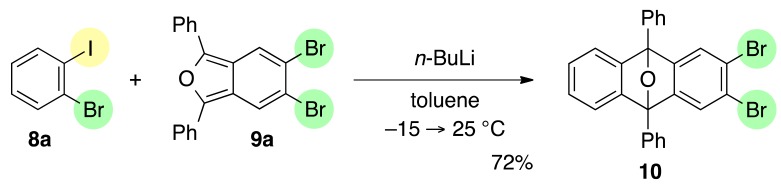
[2+4] cycloaddition of benzyne **B** and isobenzofuran **9a**.

Further study revealed that 5,6-dibromo-1,3-diphenylisobenzofuran (**9a**) was also a suitable reactive partner, which cyclized with benzyne **B**, generated by treatment of iodobromide **8a** with *n*-BuLi (toluene, −15→25 °C), affording substituted epoxyanthracene **10** in 72% yield (Scheme 4).

**Scheme 5:**
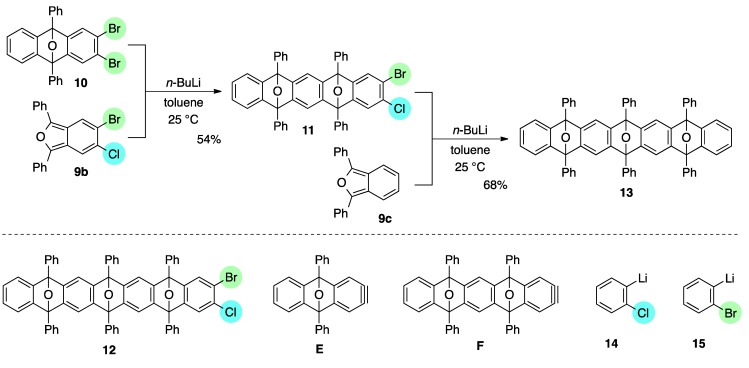
Mono-directional [2+4] cycloadditions of arynes.

We next examined second [2+4] cycloaddition of aryne generated from the first cycloadduct. To explore another mode of selective halogen-lithium exchange of 1,2-haloarenes, *i.e.*, reactivity control by adjacent halogen (type 2 in Scheme 3) [38,39], two different halogens were introduced to isobenzofuran. Upon treatment of dibromide **10** with 1.3 equiv. of *n*-BuLi in the presence of 1.1 equiv. of 5-bromo-6-chloro-1,3-diphenylisobenzofuran (**9b**) [40] (toluene, 25 °C), aryne **E** was selectively generated and subsequent trapping with **9b** gave mono-cycloadduct **11** in 54% yield as a mixture of diastereomers (Scheme 5). In this case, bis-cycloadduct **12**, caused by the generation of aryne **F**, was produced in 16% yield. This observed site-selectivity in the bromine-lithium exchange among three bromides **9b**, **10**, and **11** was unexpected, because (2-chlorophenyl)lithium **14** was more thermodynamically stable than (2-bromophenyl)lithium **15** by existing of a more electron withdrawing chlorine atom, which would suggest the favorable formation of aryne **F** over that of aryne **E** [41]. Aside from the unanticipated site-selectivity in this bromine-lithium exchange, further introduction of fused ring onto the dual cycloadduct **11** was realized by the third [2+4] cycloaddition of aryne **F** and isobenzofuran **9c** by treatment of **11** with *n*-BuLi under the similar conditions, affording polycyclic compound **13** in 66% yield, which is expected to be suitably converted to substituted heptacenes [42,43,44,45].

**Scheme 6:**
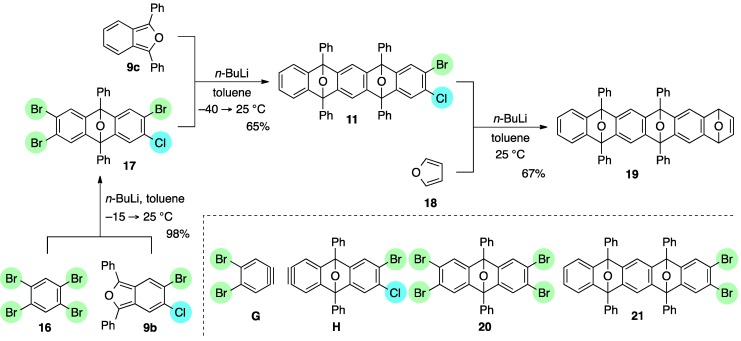
Bi-directional [2+4] cycloadditions of arynes.

Moreover, it is worth mentioning that 1,2,4,5-tetrabromobenzene (**16**) nicely worked as a reactive platform [46,47,48,49,50,51], allowing bi-directional cycloadditions in an unsymmetrical manner (Scheme 6). The essential point of this sequential process is using 5-bromo-6-chloro-1,3-diphenylisobenzofuran (**9b**) to differentiate the reactivity of the two dihalogenated sites in the bis-aryne equivalent **17**, which was efficiently obtained by the first [2+4] cycloaddition of dibromobenzyne **G** and isobenofuran **9b**. It is notable that perfect site-selectivity was observed in the bromine-lithium exchange of **16**, selectively generating the dibromobenzyne **G** [52]. The cycloadduct **17**, thus obtained, again underwent the selective bromine-lithium exchange at the dibromo side in **17**, as a related reaction of dibromide **10** and isobenzofuran **9b** (Scheme 5), generating the bromochlorobenzyne **H**, which was intercepted by **9c** to afford the unsymmetrical cycloadduct **11** in 65% yield, accompanied by a formation of dual cycloadduct **21** (20%). Although the observed selectivity in the reaction of **17** was moderate (**11**/**21** = 3.2:1), use of bis-aryne equivalent **17** with an unsymmetric form turned out to be indispensible to discriminate the reactivity of the two dihalogenated sites in **17**, because the corresponding reaction of the symmetrical tetrabromide **20** resulted in the decreased selectivity in the formation of the desired mono-cycloadduct **21** and bis-cycloadduct **13** (**21**/**13** = 1.5:1). Final [2+4] cycloaddition of aryne **F**, generated from the bis-cycloadduct **11**, with furan **18** under the above-mentioned conditions was satisfied, efficiently affording the tris-cycloadduct **19** with a various synthetic opportunity for further introduction of fused rings and/or functionalization.

## 3. Experimental Section

### General Information

All experiments dealing with air- and moisture-sensitive compounds were conducted under an atmosphere of dry argon. Toluene (anhydrous; Wako Pure Chemical Industries, Ltd., Osaka, Japan) was used as received. For thin-layer chromatography (TLC) analysis, Merck pre-coated plates (silica gel 60 F_254_, Art 5715, 0.25 mm, Merck Japan, Tokyo, Japan) were used. For flash column chromatography, silica gel 60 N (spherical, neutral, 63–210 μm) from Kanto Chemical (Tokyo, Japan) was used. Silica gel preparative TLC (PTLC) was performed on Merck silica gel 60 PF_254_ (Art 7747).

^1^H-NMR and ^13^C-NMR were measured on a JNM ECA-300 and a JNM ECX-500II spectrometer (JEOL, Tokyo, Japan). Attenuated Total Reflectance Fourier Transformation Infrared (ATR-FTIR) spectra were recorded on a FT/IR-4200 FT-IR Spectrometer (JASCO, Tokyo, Japan). High resolution mass spectra were obtained with a JEOL JMS 700 spectrometer and a JEOL AccuTOF LC-plus JMS-T100LP. Melting point (mp) determinations were performed by using a MP-S3 instrument (Yanako, Kyoto, Japan) or a MPA100 Automated Melting Point System (OptiMelt, Sunnyvale, CA, USA) and are uncorrected.

Typical Procedure for [2+4] Cycloadditions of Aryne and Isobenzofuran: Synthesis of *2,3-Dibromo-9,10-dihydro-9,10-epoxyanthracene* (**6a**). To a mixture of 1-bromo-2-iodobenzene (**8a**, 70.0 mg, 0.247 mmol) and isobenzofuran **1** (71.8 mg, 0.260 mmol) in toluene (2.0 mL) was added *n*-BuLi (1.60 M in *n*-hexane, 0.19 mL, 0.30 mmol) at −15 °C, and the reaction was warmed up to 25 °C. After 5 min, the reaction was stopped by adding water. The products were extracted with EtOAc (×3), and the combined organic extracts were washed with brine, dried (Na_2_SO_4_), and concentrated in vacuo. The residue was purified by PTLC (hexane/EtOAc = 8/2) to give 2,3-dibromo-9,10-dihydro-9,10-epoxyanthracene (**6a**, 67.9 mg, 78.1%) as a white solid and 2,3-dibromo-5,7,12,14-tetrahydro-5,14:7,12-diepoxypentacene (**7a**, 1.2 mg, 1.0%, ds: 17/83) as a mixture of diastereomers.


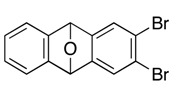


*Compound* **6a**: Mp 208.5–209.1 °C (hexane/CHCl_3_); ^1^H-NMR (CDCl_3_, δ) 6.01 (s, 2H), 7.06 (dd, 2H, *J*_1_ = 3.1 Hz, *J*_2_ = 5.2 Hz), 7.33 (dd, 2H, *J*_1_ = 3.1 Hz, *J*_2_ = 5.2 Hz), 7.55 (s, 2H); ^13^C-NMR (CDCl_3_, δ) 82.0, 120.7, 121.6, 125.7, 126.5, 146.9, 149.2; IR (ATR) 3027, 1569, 1459, 1259, 1085, 953, 832, 762 cm^−1^; ^−^HRMS (FAB) *m*/*z* 351.8925 (351.8922 calcd for C_14_H_8_Br_2_O, M^+^).


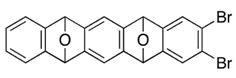


*Compound* **7a**, less polar diastereomer: R*_f_* 0.30 (hexane/CH_2_Cl_2_= 4/6); Mp decomposed at 300 °C; ^1^H-NMR (CDCl_3_, δ) 5.90 (s, 2H), 5.95 (s, 2H), 7.00 (dd, 2H, *J*_1_ = 3.1 Hz, *J*_2_ = 5.5 Hz), 7.29 (dd, 2H, *J*_1_ = 3.1 Hz, *J*_2_ = 5.5 Hz), 7.31 (s, 2H), 7.53 (s, 2H); ^13^C-NMR (CDCl_3_, δ) 81.9, 82.4, 114.2, 120.4, 121.6, 125.6, 126.0, 146.1, 147.8, 148.1, 149.2; IR (ATR) 3016, 1569, 1457, 1265, 1085, 949, 832, 772 cm^−1^; ^−^HRMS (FAB) *m*/*z* 468.9262 (468.9263 calcd for C_22_H_13_Br_2_O_2_, [M + H]^+^).

*Compound* **7a**, more polar diastereomer: R*_f_* 0.13 (hexane/CH_2_Cl_2_ = 4/6); Mp decomposed at 300 °C; ^1^H-NMR (CDCl_3_, δ) 5.90 (s, 2H), 5.96 (s, 2H), 6.97 (dd, 2H, *J*_1_ = 3.1 Hz, *J*_2_ = 5.2 Hz), 7.27 (dd, 2H, *J*_1_ = 3.1 Hz, *J*_2_ = 5.2 Hz), 7.29 (s, 2H), 7.46 (s, 2H); ^13^C-NMR (CDCl_3_, δ) 82.0, 82.5, 113.9, 120.5, 121.5, 125.6, 125.9, 146.0, 147.9, 149.2; IR (ATR) 3010, 1573, 1457, 1271, 1086, 952, 836, 754 cm^−1^; ^−^HRMS (FAB) *m*/*z* 468.9256 (468.9263 calcd for C_22_H_13_Br_2_O_2_, [M + H]^+^).

*2,3-Dibromo-9,10-diphenyl-9,10-epoxyanthracene* (**10**). According to the procedure described for the synthesis of cycloadduct **6a**, 1-bromo-2-iodobenzene (**8a**, 112 mg, 0.396 mmol), isobenzofuran **9a** (129 mg, 0.301 mmol) and *n*-BuLi (1.60 M in *n*-hexane, 0.25 mL, 0.40 mmol) gave, after purified by silica-gel flash column chromatography (hexane/CH_2_Cl_2_/Et_2_O = 98/1/1→96/3/1), cycloadduct **10** (110 mg, 72.4%) as a white solid.


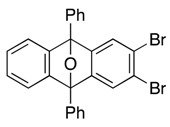


*Compound* **10**: Mp 167.6–168.5 °C (hexane/Et_2_O); ^1^H-NMR (CDCl_3_, δ) 7.08 (dd, 2H, *J*_1_ = 2.9 Hz, *J*_2_ = 5.7 Hz), 7.38 (dd, 2H, *J*_1_ = 2.9 Hz, *J*_2_ = 5.7 Hz), 7.49–7.53 (m, 2H), 7.54 (s, 2H), 7.59–7.63 (m, 4H), 7.86–7.89 (m, 4H); ^13^C-NMR (CDCl_3_, δ) 90.2, 120.7, 121.8, 125.6, 126.4, 126.5, 128.7, 129.0, 133.9, 149.2, 151.6; IR (ATR) 3030, 1599, 1499, 1295, 1036, 992, 871, 741 cm^−1^; ^−^HRMS (DART) *m*/*z* 502.9644 (502.9646 calcd for C_26_H_17_Br_2_O, [M + H]^+^).

*2-Bromo-3-chloro-5,7,12,14-tetraphenyl-5,14:7,12-diepoxypentacene* (**11**). According to the procedure described for the synthesis of cycloadduct **6a**, cycloadduct **10** (75.6 mg, 0.150 mmol), isobenzofuran **9b** (63.2 mg, 0.165 mmol) and *n*-BuLi (1.60 M in *n*-hexane, 0.12 mL, 0.19 mmol) gave, after purification by silica-gel flash column chromatography (hexane/CH_2_Cl_2_/Et_2_O = 96/3/1→88/9/3), 2-bromo-3-chloro-5,7,12,14-tetraphenyl-5,14:7,12-diepoxypentacene (**11**, 58.8 mg, 53.9%, ds. less polar/more polar = 46/54) and 2-bromo-3-chloro-5,7,12,14-tetraphenyl-5,14:7,12-diepoxy-pentacene (**12**, 23.7 mg, 15.9%) as a mixture of diastereomers, respectively. The diastereomers of **11** were separated by PTLC (hexane/toluene/CH_2_Cl_2_/Et_2_O = 82/10/6/2 X2), affording less polar **11** and more polar **11** as white solids.


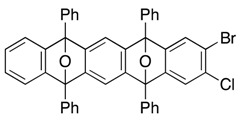


*Compound* **11**, less polar: R*_f_* 0.38 (hexane/toluene/CH_2_Cl_2_/Et_2_O = 82/10/6/2, X2); Mp decomposed at 240 °C (MeOH/CHCl_3_); ^1^H-NMR (CDCl_3_, δ) 6.96 (dd, 2H, *J*_1_ = 2.9 Hz, *J*_2_ = 5.2 Hz), 7.27 (dd, 2H, *J*_1_ = 2.9 Hz, *J*_2_ = 5.2 Hz), 7.29 (s, 1H), 7.33 (s, 2H), 7.44 (s, 1H), 7.46–7.51 (m, 4H), 7.56–7.60 (m, 8H), 7.77–7.79 (m, 4H), 7.83–7.86 (m, 4H); ^13^C-NMR (CDCl_3_, δ) 90.2, 90.3, 90.5, 113.7, 119.3, 120.4, 122.6, 125.6, 125.8, 126.4, 126.6, 128.4, 128.7, 128.9, 129.1, 131.5, 133.70, 133.73, 134.6, 148.26, 148.34, 149.9, 150.15, 150.17, 150.6, 151.3; IR (ATR) 3059, 1607, 1500, 1308, 1083, 986, 867, 744 cm^−1^; ^−^HRMS (ESI) *m*/*z* 749.0834 (749.0859 calcd for C_46_H_28_BrClNaO_2_, [M + Na]^+^).

*Compound* **11**, more polar: R*_f_* 0.28 (hexane/toluene/CH_2_Cl_2_/Et_2_O = 82/10/6/2, X2); Mp decomposed at 230 °C (MeOH/CHCl_3_); ^1^H-NMR (CDCl_3_, δ) 7.02 (dd, 2H, *J*_1_ = 2.9 Hz, *J*_2_ = 5.2 Hz), 7.30 (s, 2H), 7.32–7.35 (m, 3H), 7.43–7.49 (m, 5H), 7.52–7.57 (m, 8H), 7.75–7.77 (m, 4H), 7.81–7.84 (m, 4H); ^13^C-NMR (CDCl_3_, δ) 90.2, 90.3, 90.5, 113.49, 113.51, 119.3, 120.5, 122.7, 125.6, 125.7, 126.4, 126.5, 128.3, 128.7, 128.8, 129.0, 131.5, 133.7, 133.8, 134.6, 148.2, 148.3, 149.9, 150.0, 150.3, 150.8, 151.6; IR (ATR) 3065, 1607, 1498, 1311, 1082, 989, 863, 746 cm^−1^; ^−^HRMS (ESI) *m*/*z* 749.0876 (749.0859 calcd for C_46_H_28_BrClNaO_2_, [M + Na]^+^).


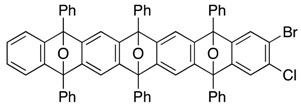


*Compound* **12** (a mixture of four diastereomers): ^1^H-NMR (CDCl_3_, δ) 6.90–7.01 (m, 8H), 7.19–7.33 (m, 24H), 7.36–7.60 (m, 80H), 7.66–7.83 (m, 48H); ^13^C-NMR (CDCl_3_, δ) 90.0, 90.05, 90.07, 90.09, 90.11, 90.14, 90.17, 90.19, 90.36, 90.39, 90.46, 90.50, 113.3, 113.4, 113.47, 113.51, 113.7, 113.8, 119.2, 119.3, 119.5, 119.6, 120.08, 120.11, 120.2, 120.4, 122.4, 122.5, 122.7, 125.4, 125.5, 125.55, 125.62, 125.7, 125.8, 125.97, 126.00, 126.07, 126.12, 126.2, 126.3, 126.42, 126.44, 126.5, 126.57, 126.64, 126.7, 128.16, 128.21, 128.3, 128.4, 128.5, 128.59, 128.63, 128.66, 128.72, 128.8, 128.86, 128.90, 128.92, 129.00, 129.03, 131.4, 131.5, 131.58, 131.63, 133.68, 133.72, 133.76, 133.83, 133.87, 133.90, 134.3, 134.38, 134.44, 134.70, 134.73, 134.78, 134.80, 147.99, 148.03, 148.1, 148.2, 148.26, 148.30, 148.4, 148.9, 148.98, 149.02, 149.1, 149.2, 149.4, 149.49, 149.52, 149.70, 149.73, 149.78, 149.80, 149.86, 149.94, 149.96, 150.02, 150.2, 150.3, 150.35, 150.42, 150.5, 150.6, 151.18, 151.24, 151.37, 151.39; IR (ATR) 3062, 1606, 1499, 1307, 1082, 983, 885, 748 cm^−1^; ^−^HRMS (ESI) *m*/*z* 1017.1756 (1017.1747 calcd for C_66_H_40_BrClNaO_3_, [M + Na]^+^).

*5,7,9,14,16,18-Hexaphenyl-5,18:7,16:9,14-triepoxyheptacene* (**13**). According to the procedure described for the synthesis of cycloadduct **6a**, cycloadduct **11** (more polar) (35.1 mg, 0.0482 mmol), isobenzofuran **9c** (14.7 mg, 0.0544 mmol) and *n*-BuLi (1.60 M in *n*-hexane, 0.040 mL, 0.064 mmol) gave, after purification by silica-gel flash column chromatography (hexane/CH_2_Cl_2_/Et_2_O = 96/3/1→88/9/3), 5,7,9,14,16,18-hexaphenyl-5,18:7,16:9,14-triepoxyheptacene (**13**) as a mixture of diastereomers (29.0 mg, 68.0%, ds. less polar/more polar = 46/54). Those diastereomers were separated by PTLC (hexane/toluene/CH_2_Cl_2_/Et_2_O = 78/10/8/4, X4), affording less polar **13** and more polar **13** as white solids, respectively.


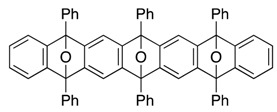


*Compound* **13** (less polar): R*_f_* 0.55 (hexane/toluene/CH_2_Cl_2_/Et_2_O = 78/10/8/4, X4); Mp decomposed at 260 °C (MeOH/CHCl_3_); ^1^H-NMR (CDCl_3_, δ); 6.92 (dd, 4H, *J*_1_ = 2.9 Hz, *J*_2_ = 5.2 Hz), 7.22 (s, 4H), 7.23 (dd, 4H, *J*_1_ = 2.9 Hz, *J*_2_ = 5.2 Hz), 7.44–7.49 (m, 6H), 7.52–7.56 (m, 12H), 7.72–7.75 (m, 4H), 7.79–7.82 (m, 8H); ^13^C-NMR (CDCl_3_, δ) 90.4, 113.4, 120.3, 125.6, 126.5, 126.6, 128.2, 128.3, 128.8, 128.9, 134.6, 134.8, 149.2, 149.4, 150.1; IR (ATR) 3058, 1603, 1496, 1307, 974, 867, 747 cm^−1^; ^−^HRMS (ESI) *m*/*z* 905.3020 (905.3032 calcd for C_66_H_42_NaO_3_, [M + Na]^+^).

*Compound* **13** (more polar): R*_f_* 0.49 (hexane/toluene/CH_2_Cl_2_/Et_2_O = 78/10/8/4, X4); Mp decomposed at 250 °C (MeOH/CHCl_3_); ^1^H-NMR (CDCl_3_, δ) 6.94 (dd, 2H, *J*_1_ = 2.9 Hz, *J*_2_ = 5.2 Hz), 6.96 (dd, 2H, *J*_1_ = 2.9 Hz, *J*_2_ = 5.2 Hz), 7.19 (s, 2H), 7.22–7.25 (m, 4H), 7.28 (s, 2H), 7.39–7.59 (m, 18H), 7.72–7.74 (m, 4H), 7.77–7.80 (m, 4H), 7.81–7.83 (m, 4H); ^13^C-NMR (CDCl_3_, δ) 90.37, 90.39, 90.5, 113.2, 113.5, 120.2, 120.3, 125.7, 125.8, 126.4, 126.5, 126.6, 128.11, 128.13, 128.3, 128.6, 128.8, 134.5, 134.8, 134.9, 149.0, 149.1, 149.3, 149.6, 149.9, 150.1; IR (ATR) 3063, 1602, 1497, 1308, 977, 869, 747 cm^−1^; ^−^HRMS (ESI) *m*/*z* 905.3028 (905.3032 calcd for C_66_H_42_NaO_3_, [M + Na]^+^).

*2,3,6-Tribromo-7-chloro-9,10-diphenyl-9,10-epoxyanthracene* (**17**). According to the procedure described for the synthesis of cycloadduct **6a**, 1,2,4,5-tetrabromobenzene (**16**, 1.54 g, 3.91 mmol), isobenzofuran **9b** (1.00 g, 2.61 mmol) and *n*-BuLi (1.60 M in *n*-hexane, 2.50 mL, 4.00 mmol) gave, after purification by silica-gel flash column chromatography (hexane/CH_2_Cl_2_/Et_2_O = 96/3/1), 2,3,6-tribromo-7-chloro-9,10-diphenyl-9,10-epoxyanthracene (**17**, 1.58 g, 98.1%) as a white solid.


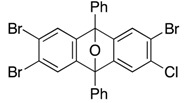


*Compound* **17**: Mp 247.2–248.0 °C (hexane/CHCl_3_); ^1^H-NMR (CDCl_3_, δ) 7.41 (s, 1H), 7.52–7.56 (m, 2H), 7.559 (s, 1H), 7.564 (s, 2H), 7.61–7.65 (m, 4H), 7.80–7.83 (m, 4H); ^13^C-NMR (CDCl_3_, δ) 89.8, 90.0, 120.1, 122.5, 122.9, 125.9, 126.3, 129.1, 129.2, 132.3, 132.89, 132.92, 149.5, 150.3, 150.37, 150.44; IR (ATR) 3017, 1601, 1499, 1288, 1089, 987, 887, 746 cm^−1^; ^−^HRMS (DART) *m*/*z* 614.8381 (614.8362 calcd for C_26_H_15_Br_3_ClO, [M + H]^+^).

*2-Bromo-3-chloro-5,7,12,14-tetraphenyl-5,14:7,12-diepoxypentacene* (**11**). According to the procedure described for the synthesis of cycloadduct **6a**, cycloadduct **17** (124 mg, 0.201 mmol), isobenzofuran **9c** (59.7 mg, 0.221 mmol) and *n*-BuLi (1.60 M in *n*-hexane, 0.15 mL, 0.24 mmol) gave, after purification by silica-gel flash column chromatography (hexane/CH_2_Cl_2_/Et_2_O = 96/3/1→88/9/3), cycloadduct **11** (94.3 mg, 64.7%, ds. less polar/more polar = 52/48) and cycloadduct **13** as a mixture of diastereomers (33.6 mg, 20.0%), respectively.

*1,4-Dihydro-6,8,13,15-tetraphenyl-1,4:6,15:8,13-triepoxyhexacene* (**19**). According to the procedure described for the synthesis of cycloadduct **6a**, cycloadduct **11** (less polar) (67.9 mg, 0.0933 mmol), furan **18** (65 mg, 0.96 mmol) and *n*-BuLi (1.63 M in *n*-hexane, 0.075 mL, 0.12 mmol) gave, after purification by PTLC (hexane/CH_2_Cl_2_/acetone = 7/2/1), 1,4-dihydro-6,8,13,15-tetraphenyl-1,4:6,15:8,13-triepoxyhexacene (**19**) as a mixture of diastereomers (42.3 mg, 66.6%).


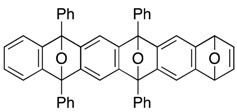


*Compound* **19** (a mixture of two diastereomers): ^1^H-NMR (CDCl_3_, δ) 5.50 (s, 2H), 5.52 (s, 2H), 6.88 (s, 2H), 6.91 (s, 2H), 6.92–6.95 (m, 4H), 7.13 (s, 2H), 7.19 (s, 2H), 7.23 (dd, 2H, *J*_1_ = 2.9 Hz, *J*_2_ = 5.2 Hz), 7.26 (dd, 2H, *J*_1_ = 2.9 Hz, *J*_2_ = 5.2 Hz), 7.29 (s, 2H), 7.33 (s, 2H), 7.44–7.49 (m, 8H), 7.54–7.59 (m, 16H), 7.79–7.87 (m, 16H); ^13^C-NMR (CDCl_3_, δ) 82.2, 82.3, 90.47, 90.52, 113.40, 113.43, 113.6, 113.8, 120.2, 120.4, 125.6, 125.7, 126.5, 126.55, 126.60, 126.9, 128.3, 128.76, 128.81, 134.8, 134.9, 143.19, 143.22, 148.2, 148.3, 148.6, 148.8, 149.2, 149.5, 149.8, 150.0, 150.2; IR (ATR) 3062, 1602, 1499, 1308, 984, 848, 744, 700 cm^−1^; ^−^HRMS (ESI) *m*/*z* 703.2233 (703.2249 calcd for C_50_H_32_NaO_3_, [M + Na]^+^).

*2,3-Dibromo-5,7,12,14-tetraphenyl-5,14:7,12-diepoxypentacene* (**21**). According to the procedure described for the synthesis of cycloadduct **6a**, 2,3,6,7-tetrabromo-9,10-diphenyl-9,10-epoxyanthracene (**20**, 110 mg, 0.166 mmol) and isobenzofuran **9c** (49.5 mg, 0.183 mmol) and *n*-BuLi (1.63 M in *n*-hexane, 0.12 mL, 0.20 mmol) gave, after purification by silica-gel flash column chromatography (hexane/CH_2_Cl_2_/Et_2_O = 96/3/1→88/9/3), *2,3-dibromo-5,7,12,14-tetraphenyl-5,14:7,12-diepoxypentacene* (**21**, 45.4 mg, 35.4%, ds. less polar/more polar = 48/52) and *2,3-dibromo-5,7,12,14-tetraphenyl-5,14:7,12-diepoxypentacene* (**13**) as a mixture of diastereomers (35.0 mg, 23.9%), respectively.


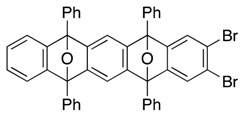


*Compound* **21** (less polar): R*_f_* 0.62 (hexane/toluene/CH_2_Cl_2_/Et_2_O = 82/10/6/2, X3); Mp decomposed at 250 °C (MeOH/CHCl_3_); ^1^H-NMR (CDCl_3_, δ) 6.96 (dd, 2H, *J*_1_ = 2.9 Hz, *J*_2_ = 5.2 Hz), 7.27 (dd, 2H, *J*_1_ = 2.9 Hz, *J*_2_ = 5.2 Hz), 7.33 (s, 2H), 7.44 (s, 2H), 7.46–7.51 (m, 4H), 7.56–7.60 (m, 8H), 7.76–7.79 (m, 4H), 7.83–7.86 (m, 4H); ^13^C-NMR (CDCl_3_, δ) 90.2, 90.5, 113.7, 120.4, 121.7, 125.6, 125.8, 126.4, 126.6, 128.4, 128.7, 128.9, 129.0, 133.7, 134.6, 148.3, 149.9, 150.2, 151.4; IR (ATR) 3059, 1606, 1499, 1308, 1033, 984, 866, 743 cm^−1^; ^−^HRMS (ESI) *m*/*z* 793.0333 (793.0354 calcd for C_46_H_28_Br_2_NaO_2_, [M + Na]^+^).

*Compound* **21** (more polar): R*_f_* 0.52 (hexane/toluene/CH_2_Cl_2_/Et_2_O = 82/10/6/2, X3); Mp decomposed at 250 °C (MeOH/CHCl_3_); ^1^H-NMR (CDCl_3_, δ) 7.01 (dd, 2H, *J*_1_ = 2.9 Hz, *J*_2_ = 5.2 Hz), 7.31 (s, 2H), 7.33 (dd, 2H, *J*_1_ = 2.9 Hz, *J*_2_ = 5.2 Hz), 7.42–7.48 (m, 4H), 7.482 (s, 2H), 7.52–7.57 (m, 8H), 7.75–7.77 (m, 4H), 7.81–7.84 (m, 4H); ^13^C-NMR (CDCl_3_, δ) 90.2, 90.5, 113.5, 120.5, 121.7, 125.70, 125.74, 126.4, 126.6, 128.3, 128.7, 128.8, 129.0, 133.7, 134.7, 148.2, 150.0, 150.3, 151.7; IR (ATR) 3063, 1601, 1499, 1310, 1032, 983, 862, 745 cm^−1^; ^−^HRMS (ESI) *m*/*z* 793.0361 (793.0354 calcd for C_46_H_28_Br_2_NaO_2_, [M + Na]^+^).

## 4. Conclusions

Site-selective halogen-lithium exchange of 1,2-dihaloarenes allowed for the successive generation of benzynes and subsequent multiple [2+4] cycloadditions with various arynophiles to give highly functionalized polycyclic compounds, which were amenable to selective transformation en route to substituted polyacene derivatives. Further synthetic applications are under active investigation in our laboratories.

## Supplementary Materials

Supplementary materials can be accessed at: http://www.mdpi.com/1420-3049/20/10/19449/s1.
