# Supplementary material for: Selective Halogen-Lithium Exchange of 1,2-Dihaloarenes for Successive [2+4] Cycloadditions of Arynes and Isobenzofurans
Source: Molecules. 2015 Oct 23;20(10):19449–62. doi: 10.3390/molecules201019449 (PMC6331892; doi:10.3390/molecules201019449)
Supplement: Supplementary file 1 [file molecules-20-19449-s001.pdf]

Supplementary Materials

1. General Experimental Procedures

All experiments dealing with air- and moisture-sensitive compounds were conducted under an atmosphere of dry argon. THF (anhydrous; Wako Pure Chemical Industries, Ltd., Osaka, Japan) was used as received.

For thin-layer chromatography (TLC) analysis, Merck pre-coated plates (silica gel 60 F_{254}, Art 5715, 0.25 mm, Merck Japan, Tokyo, Japan) were used. For flash column chromatography, silica gel 60 N (spherical, neutral, 63–210 μm) from Kanto Chemical (Tokyo, Japan) was used.

^{1}H-NMR and ^{13}C-NMR were measured on a JEOL JNM ECA-300 and a JEOL JNM ECX-500II spectrometer (JEOL, Tokyo, Japan). Attenuated Total Reflectance Fourier Transformation Infrared (ATR-FTIR) spectra were recorded on a FT/IR-4200 FT-IR Spectrometer (JASCO, Tokyo, Japan). High resolution mass spectra were obtained with a JEOL JMS 700 spectrometer and a JEOL AccuTOF LC-plus JMS-T100LP. Melting point (mp) determinations were performed by using a MPA100 OptiMelt Automated Melting Point System (OptiMelt, Sunnyvale, California, CA, USA) and are uncorrected.

2. Preparation of 5-Bromo-6-chloro-1,3-diphenylisobenzofuran (9b)

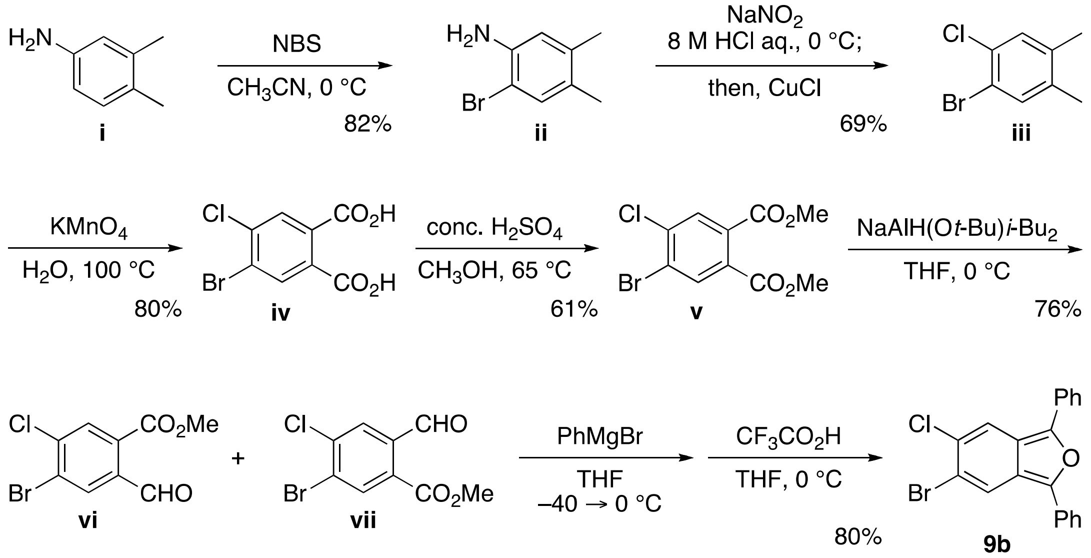

2-Bromo-4,5-dimethylaniline (**ii**). To a solution of 3,4-dimethylaniline (**i**) (30.3 g, 250 mmol) in CH_3CN (250 mL) was added *N*-bromosuccinimide (48.9 g, 275 mmol) in CH_3CN (350 mL) through a dropping funnel at 0 °C. After stirring for 0.5 h, the reaction was stopped by adding sat. aq. $NaHCO_3$. The products were extracted with EtOAc (X3), washed with brine, dried (Na_2SO_4), and concentrated *in vacuo*. The residue was purified by silica-gel flash columun chromatography (hexane/EtOAc = 95/5) to give 2-bromo-4,5-dimethylaniline (**ii**) (41.0 g, 82.0%) as white solids. Recrystallization from hexane gave **ii** as colorless prisms.

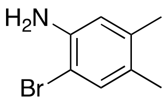

2-Bromo-4,5-dimethylaniline (**ii**). Mp 84.3–85.3 °C (hexane); ^{1}H-NMR ($CDCl_3$, δ) 2.12 (m, 6H), 3.85 (br s, 2H), 6.56 (s, 1H), 7.15 (s, 1H); ^{13}C-NMR ($CDCl_3$, δ)18.5, 19.4, 106.1, 117.2, 128.0, 132.9, 136.8, 141.7;IR (ATR) 3453, 3369, 3020, 2979, 2915, 2878, 1609, 1496, 1450, 1383, 1281, 1150, 970, 874, 853, 731 cm^{-1}; HRMS (ESI) *m*/*z* 200.0082 (200.0075 calcd for $C_8H_{11}BrN$, [M + H]$^+$).

1-Bromo-2-chloro-4,5-dimethylbenzene (**iii**). To a suspension of aniline **ii** (20.0 g, 100 mmol) in H_2O (50 mL) was added 12 M HCl (100 mL) and the reaction mixture was cooled to 0 °C, to which was gradually added $NaNO_2$ (8.99 g, 130 mmol) in H_2O (30 mL) followed by CuCl (12.1 g, 122 mmol) dissolved in 12 M HCl (30 mL) at same temperature. After warmed up to room temperature, the reaction was stirred for further 20 min. The products were extracted with Et_2O (X3), and the combined organic extracts were washed with 25% NH_3 aq. and brine, dried (Na_2SO_4), and concentrated *in vacuo*. Purification of the crude products by recrystallization from MeOH gave 1-bromo-2-chloro-4,5-dimethylbenzene (**iii**) (15.2 g, 69.2%) as colorless prisms.

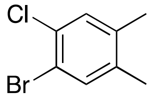

1-Bromo-2-chloro-4,5-dimethylbenzene (**iii**). Mp 74.1–74.6 °C; ^{1}H-NMR ($CDCl_3$, δ) 2.19 (s, 3H), 2.20 (s, 3H), 7.21 (s, 1H), 7.36 (s, 1H); ^{13}C-NMR ($CDCl_3$, δ) 19.0, 19.2, 118.7, 131.0, 134.2, 136.9, 137.4; IR (ATR) 3044, 2978, 2921, 2857, 1590, 1469, 1444, 1382, 1349, 1161, 1123, 1020, 993, 912, 875 cm^{-1}; HRMS (DART) *m*/*z* 217.9508 (217.9498 calcd for C_8H_8BrCl, M$^+$).

4-Bromo-5-chlorophthalic acid (**iv**). A mixture of *o*-xylene **iii** (8.50 g, 38.7 mmol) and $KMnO_4$ (30.8 g, 195 mmol) in H_2O (300 mL) was refluxed for 20 h. After cooled to room temperature, the reaction was stopped by adding $NaHSO_3$ followed by aq. KOH. The mixture was filtered through a Büchner funnel and the filtrate was acidified by adding 12 M HCl. The filtration was washed by haxane (X3) to give essentially pure 4-bromo-5-chlorophthalic acid (**iv**) (8.60 g, 79.6%) as white solids. Recrystallization from hexane/acetone gave **iv** as white solids.

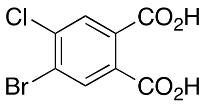

4-Bromo-5-chlorophthalic acid (**iv**). Mp decomposed at 300 °C; ^{1}H-NMR (acetone-d_6, δ) 7.91 (s, 1H), 8.08 (s, 1H); ^{13}C-NMR (acetone-d_6, δ) 125.2, 131.2, 133.5, 134.4, 134.9, 137.4, 166.7, 166.9; IR (ATR) 3099, 2812 (br), 1685, 1613, 1526, 1442, 1354, 1271, 1249, 1150, 1091, 919, 896, 759 cm^{-1}; HRMS (DART) *m*/*z* 278.9063 (278.9060 calcd for $C_8H_5BrClO_4$, [M + H]$^+$).

Dimethyl 4-bromo-5-chlorophthalate (**v**). To a solution of phthalic acid **iv** (5.60 g, 20.0 mmol) in MeOH (65 mL) was added conc. H_2SO_4 (1.6 mL, 30.0 mmol) at room temperature, and the reaction was refluxed for 5 h. After evaporating the organic solvent, sat. aq. $NaHCO_3$ was added to the reaction mixture. The products were extracted with EtOAc (X3), and the combined organic extracts were washed with brine, dried (Na_2SO_4), and concentrated *in vacuo*. The residue was purified by silica-gel flash column chromatography

(EtOAc) to give dimethyl 4-bromo-5-chlorophthalate (**v**) (3.76 g, 61.0%). Recrystallization from hexane gave **v** as colorless prisms.

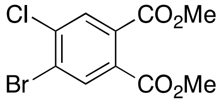

Dimethyl 4-bromo-5-chlorophthalate (**v**). Mp 45.4–45.9 °C; ^{1}H-NMR ($CDCl_3$, δ) 3.91 (m, 6H), 7.80 (s, 1H), 7.99 (s, 1H); ^{13}C-NMR ($CDCl_3$, δ) 53.1, 125.7, 130.6, 131.2, 132.1, 134.2, 137.9, 165.9, 166.1; IR (ATR) 3014, 2961, 2850, 1731, 1588, 1544, 1434, 1356, 1275, 1222, 1121, 1083, 972, 909, 876, 820, 784, 753 cm^{-1}; HRMS (ESI) *m*/*z* 306.9376 (306.9373 calcd for $C_{10}H_9BrClO_4$, $[M + H]^+$).

Methyl 4-bromo-5-chloro-2-formylbenzoate (**vi**). To a solution of dimethyl phthalate **v** (3.89 g, 12.6 mmol) in THF (25 mL) was added SDBBA [1] (0.5 M in THF, 39.0 mL, 19.5 mmol) at 0 °C. After 2.5 h, the reaction was stopped by adding 2 M HCl. The products were extracted with EtOAc (X3), and the combined organic extracts were washed with sat. aq. $NaHCO_3$ and brine, dried (Na_2SO_4), and concentrated *in vacuo*. The residue was purified by silica-gel flash column chromatography (hexane/EtOAc = 9/1) to give methyl 4-bromo-5-chloro-2-formylbenzoate (**vi**) (2.65 g, 75.8%, **vi**/**vii** = 50/50) as a mixture of regioisomers.

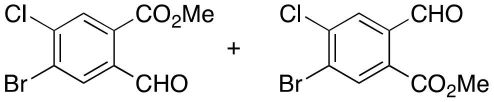

Methyl 4-bromo-5-chloro-2-formylbenzoate (**vi**) and *Methyl 5-bromo-4-chloro-2-formylbenzoate* (**vii**). ^{1}H-NMR ($CDCl_3$, δ) 3.99 (s, 6H), 8.00 (s, 1H), 8.08 (s, 1H), 8.20 (s, 1H), 8.28 (s, 1H), 10.59 (s, 1H), 10.60 (s, 1H); ^{13}C-NMR ($CDCl_3$, δ) 53.2, 128.0, 130.0, 130.6, 131.4, 132.3, 133.8, 136.0, 136.8, 139.8, 139.9, 164.7, 164.9, 189.7, 190.0; IR (ATR) 3093, 3023, 2960, 2911, 1712, 1693, 1577, 1547, 1433, 1290, 1273, 1195, 1171, 1095, 960, 916, 903, 837, 780 cm^{-1}; HRMS (DART) *m*/*z* 276.9267 (276.9267 calcd for $C_9H_7BrClO_3$, $[M + H]^+$).

5-Bromo-6-chloro-1,3-diphenylisobenzofuran (**9b**). To a solution of methyl 2-formylbenzoate **vi** and **vii** (2.46 g, 8.86 mmol) in THF (15 mL) was added phenylmagnesium bromide (1.0 M in THF, 26.0 mL, 26 mmol) at –40 °C. After warmed up to 0 °C, CF_3CO_2H (5.06 g, 44.4 mmol) was added to the mixture at same temperature, and the reaction stirred for further 10 min. The reaction was stopped by adding sat. aq. $NaHCO_3$, and the products were extracted with CH_2Cl_2 (X3), washed with brine, dried (Na_2SO_4), and concentrated *in vacuo*. Trituration of the crude products with Et_2O at −78 °C gave 5-bromo-6-chloro-1,3-diphenylisobenzofuran (**9b**) (2.73 g, 80.4%). Recrystallization from hexane/$CHCl_3$ gave **9b** as yellow needles.

---

[1] Sodium diisobutyl-*tert*-butoxyalminium (SDBBA) was prepared as follows: to a solution of sodium *tert*-butoxide (2.44 g, 25.4 mmol) in THF (25 mL) was added diisobutylalminium hydride (1.0 M in hexane, 25.0 mL, 25.0 mmol) at 0 °C, and the mixture was stirred for 1 h to give SDBBA (0.5 M in THF).

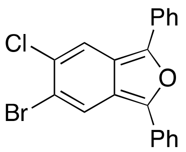

5-Bromo-6-chloro-1,3-diphenylisobenzofuran (**9b**). Mp 169.3–170.0 °C (hexane/$CHCl_3$); ^{1}H-NMR ($CDCl_3$, δ) 7.30–7.38 (m, 2H), 7.47–7.53 (m, 4H), 7.84–7.89 (m, 4H), 7.96 (s, 1H), 8.18 (s, 1H); ^{13}C-NMR ($CDCl_3$, δ) 119.7, 120.5, 120.7, 121.0, 124.8, 124.88, 124.92, 127.69, 127.74, 129.1, 130.6, 130.8; IR (ATR) 3059, 1599, 1524, 1484, 1443, 1339, 1173, 1065, 967, 900, 856, 797, 753 cm^{-1}; HRMS (DART) *m*/*z* 382.9831 (382.9838 calcd for $C_{20}H_{13}BrClO$, $[M + H]^+$).

3. ^{1}H- and ^{13}C-NMR Spectra of the Products

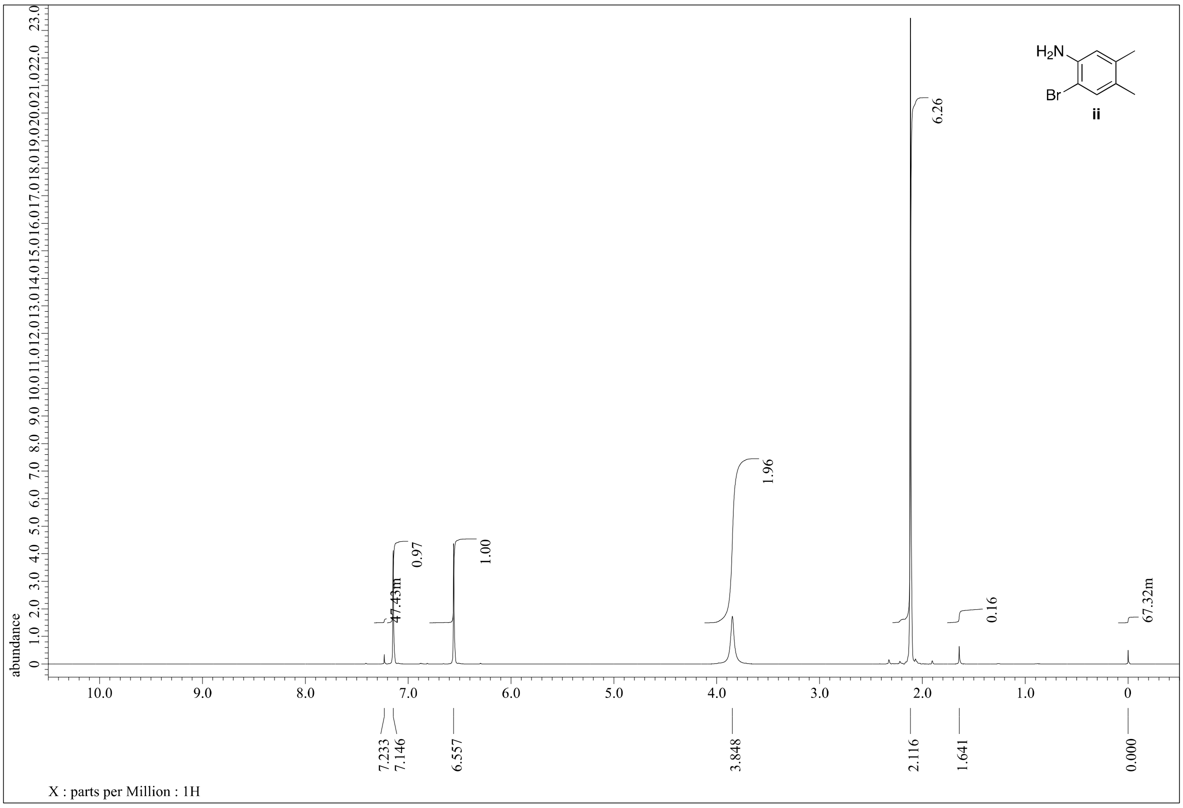

Figure S1. ^{1}H-NMR (300 MHz, $CDCl_3$) spectrum of aniline **ii**.

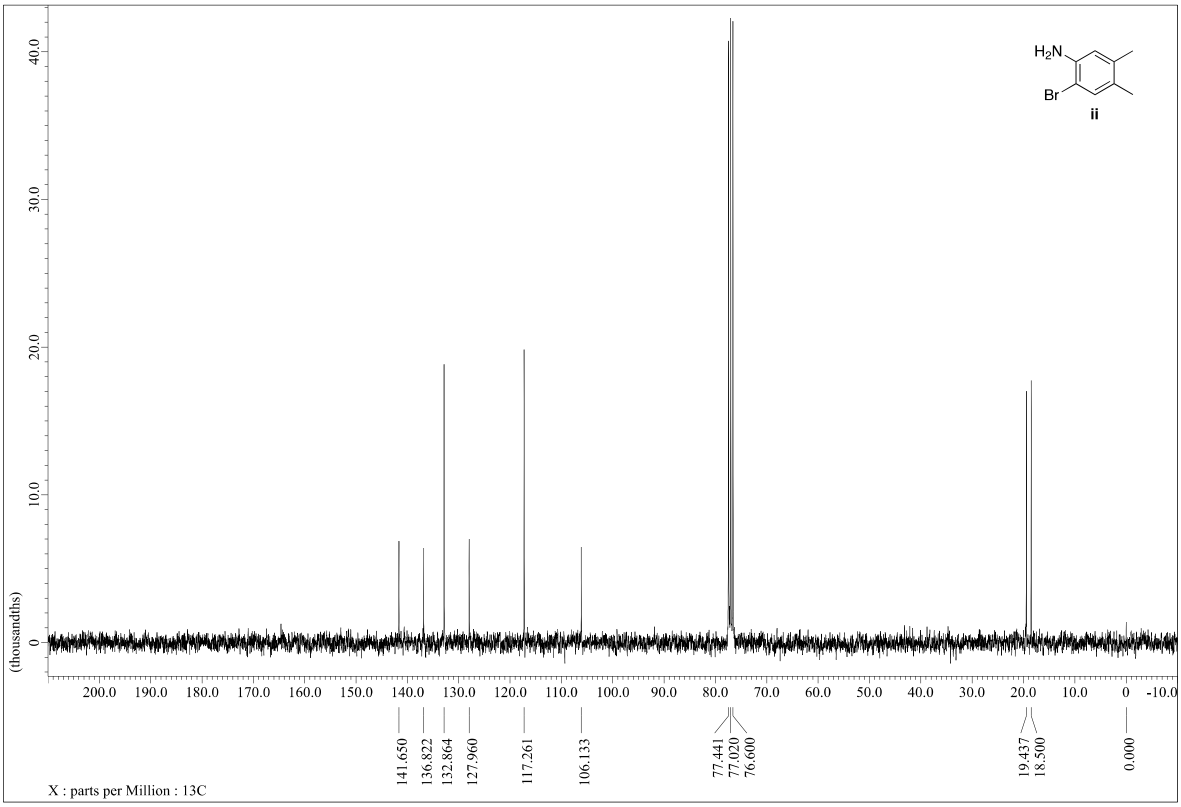

Figure S2. ^{13}C-NMR (300 MHz, $CDCl_3$) spectrum of aniline **ii**.

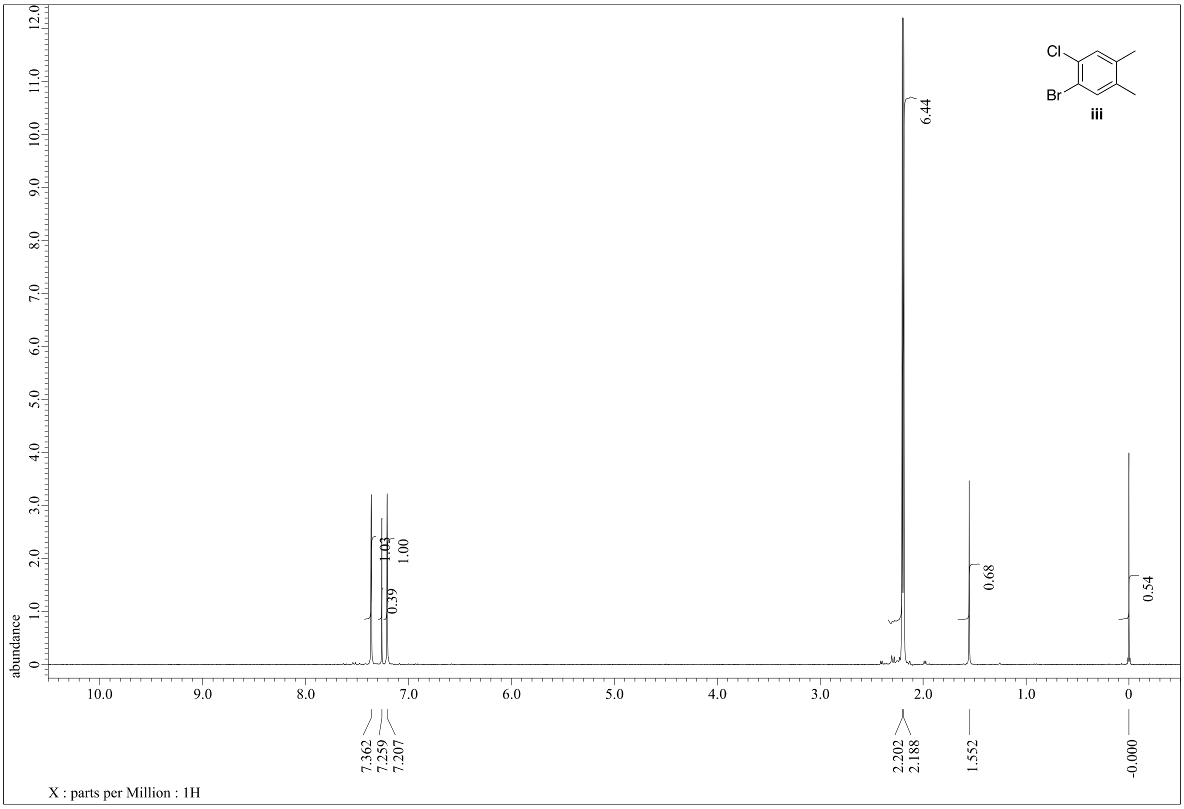

Figure S3. ^{1}H-NMR (300 MHz, $CDCl_3$) spectrum of *o*-xylene **iii**.

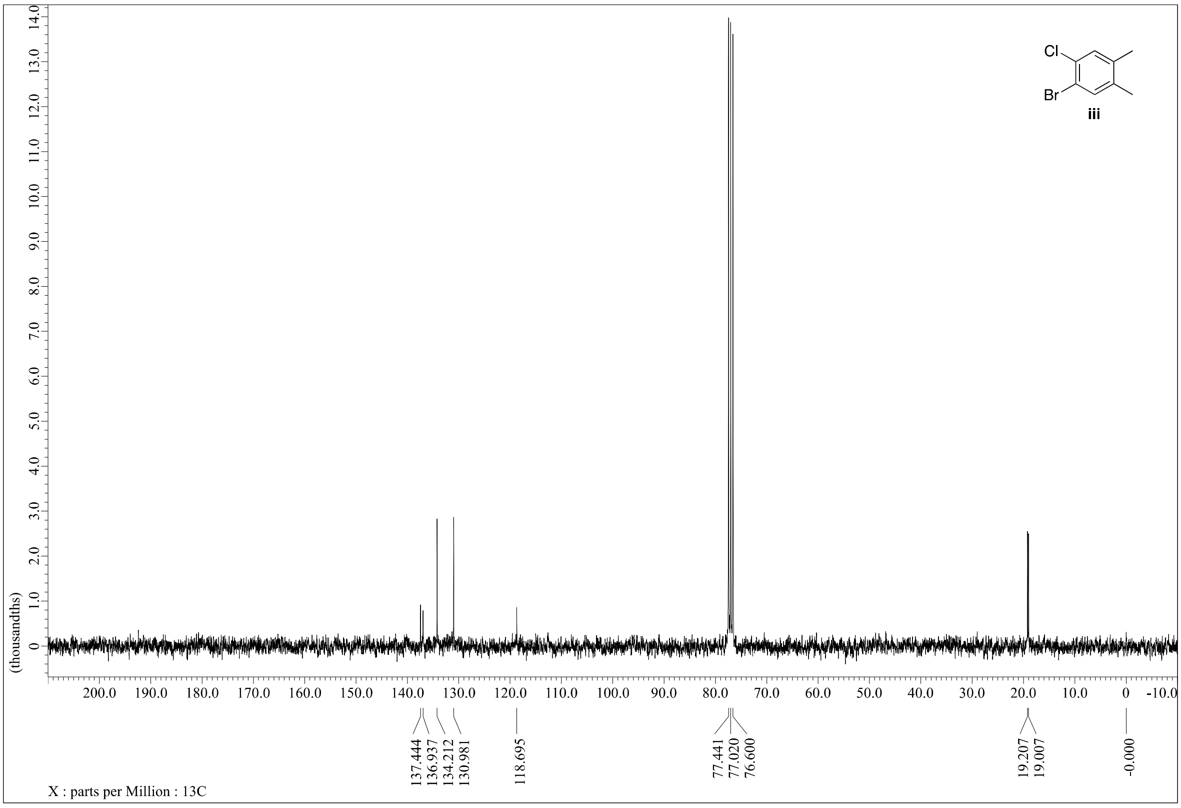

Figure S4. ^{13}C-NMR (300 MHz, $CDCl_3$) spectrum of *o*-xylene **iii**.

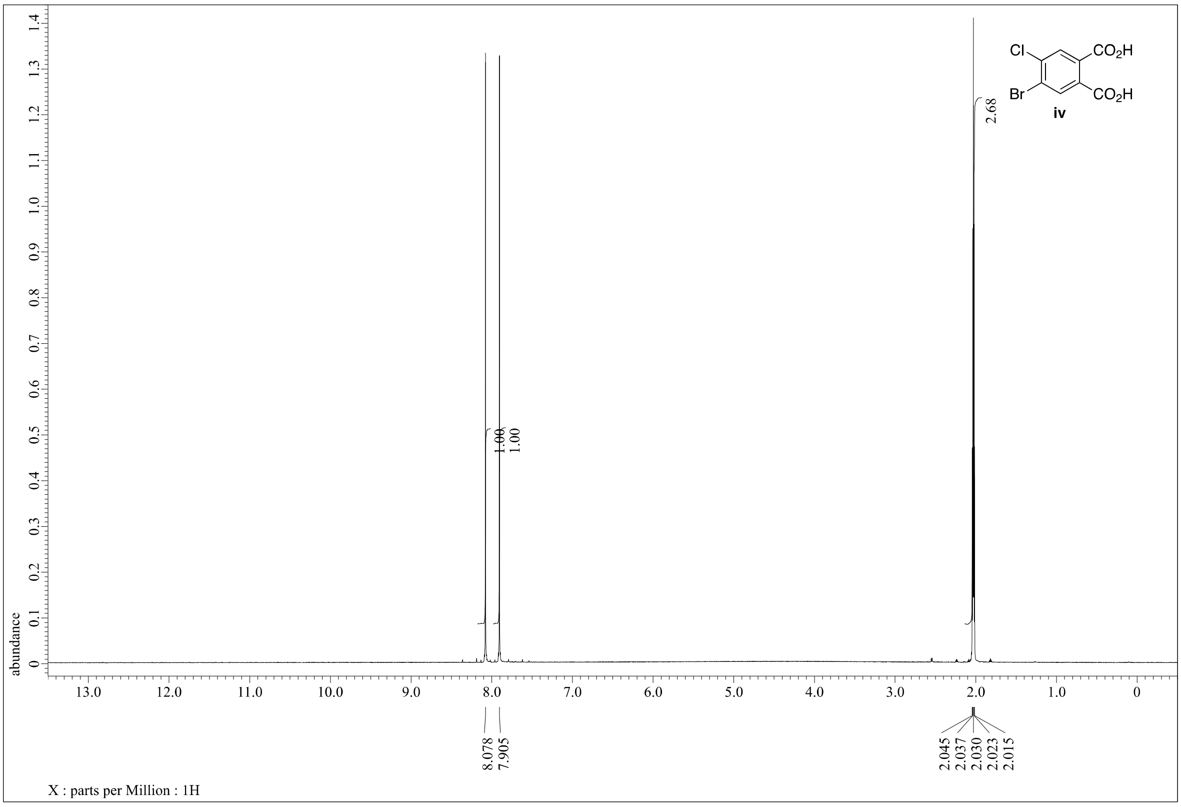

Figure S5. ^{1}H-NMR (300 MHz, acetone-d_6) spectrum of phthalic acid **iv**.

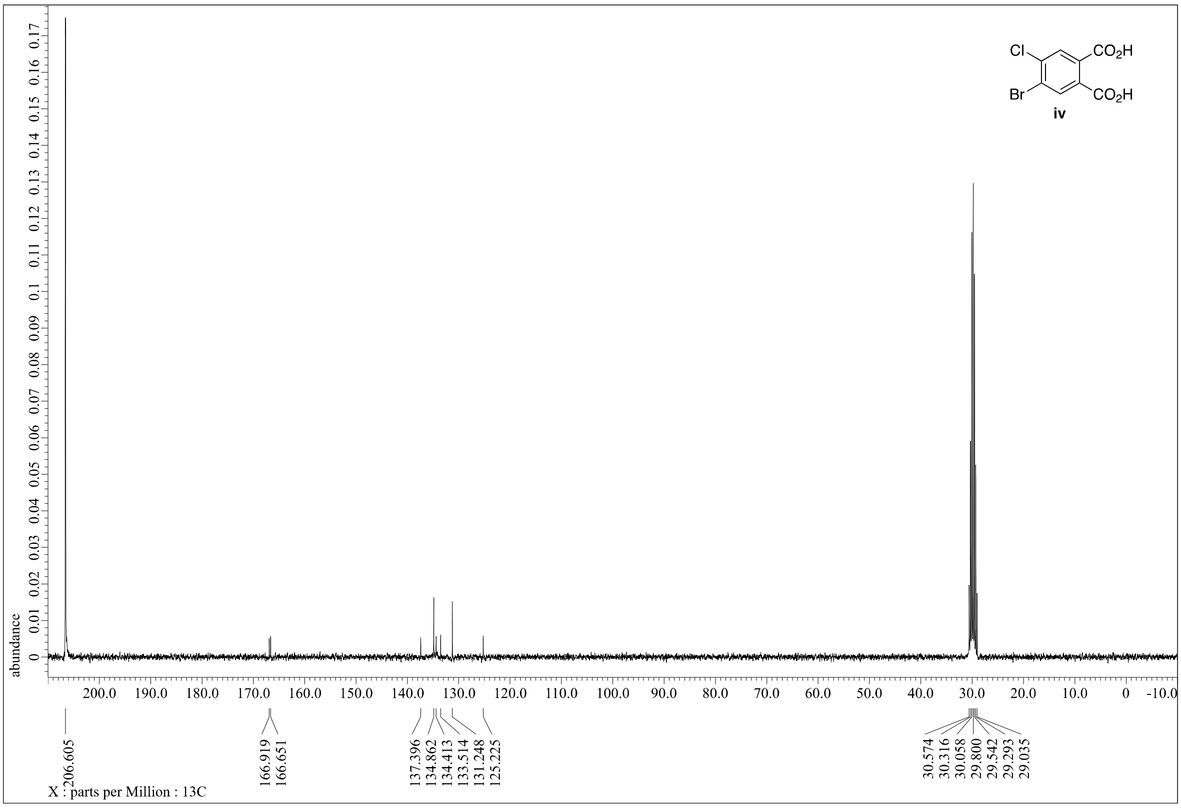

Figure S6. ^{13}C-NMR (300 MHz, acetone-d_6) spectrum of phthalic acid **iv**.

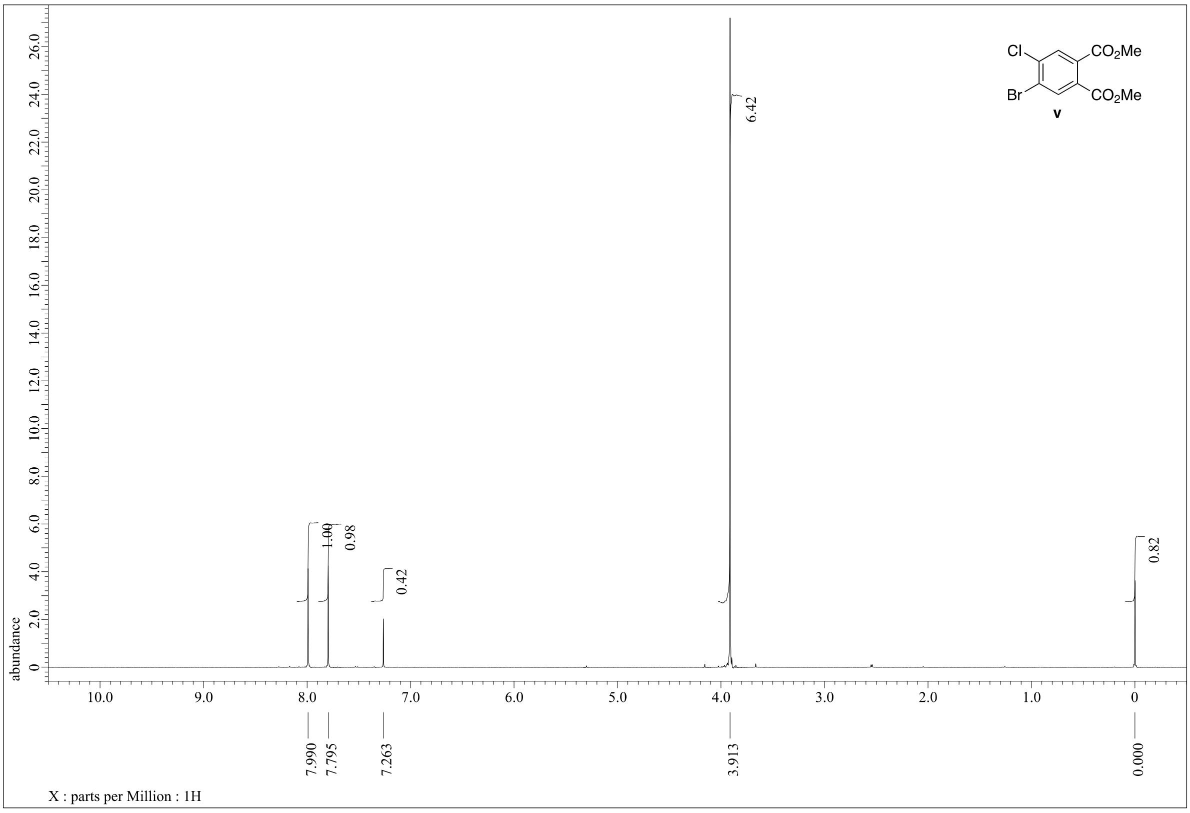

Figure S7. ^{1}H-NMR (300 MHz, $CDCl_3$) spectrum of dimethyl phthalate **v**.

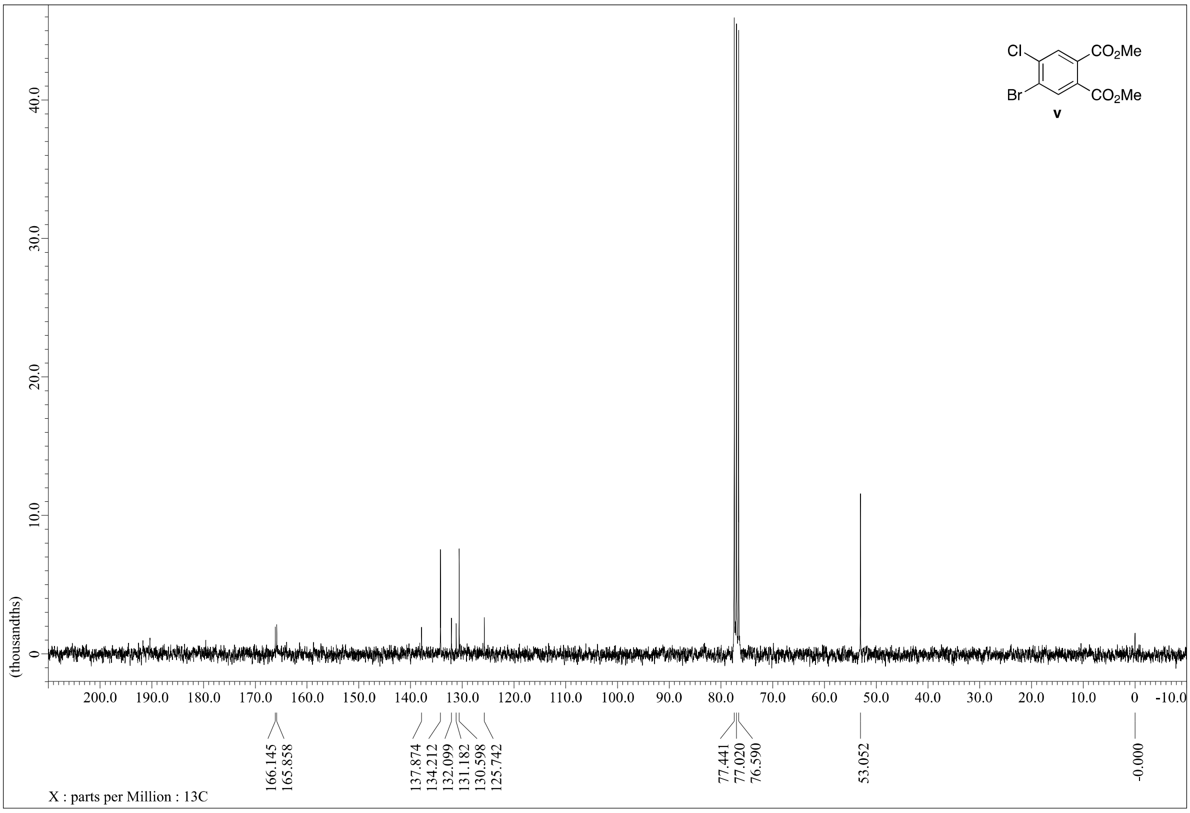

Figure S8. ^{13}C-NMR (300 MHz, $CDCl_3$) spectrum of dimethyl phthalate **v**.

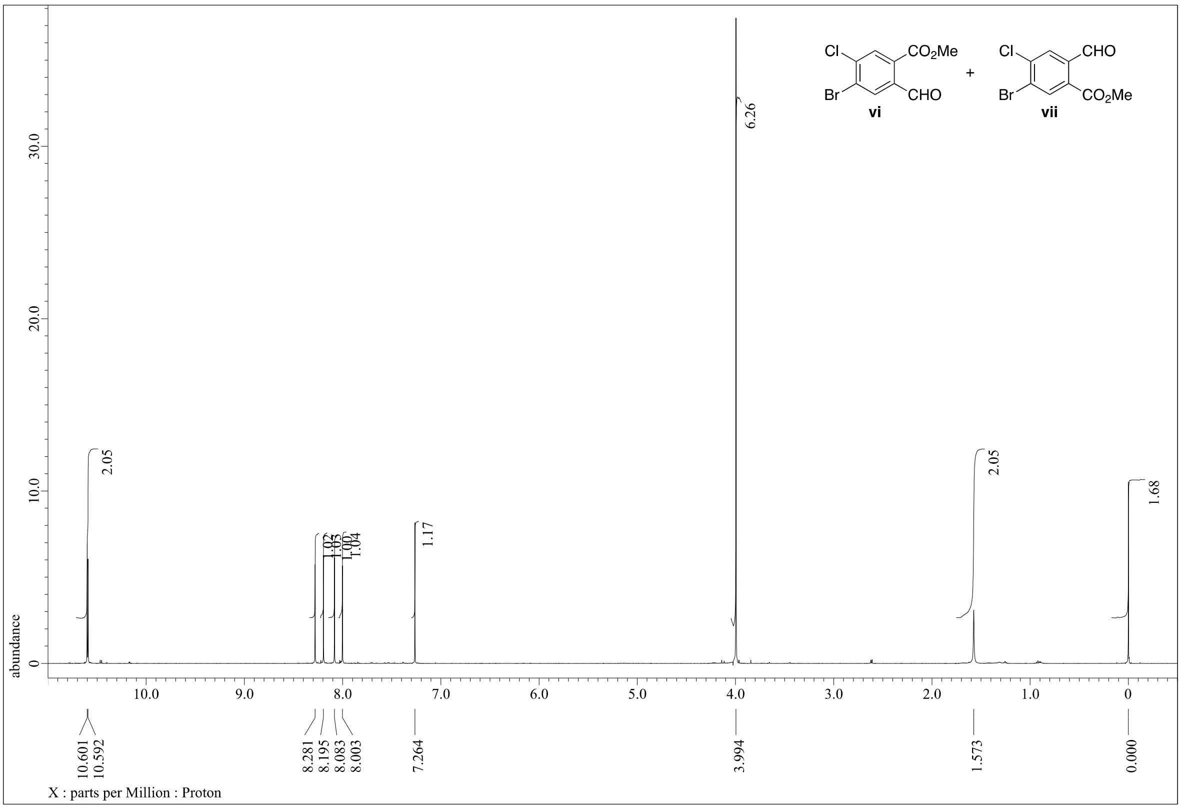

Figure S9. ^{1}H-NMR (50 0 MHz, $CDCl_3$) spectrum of methyl 2-formylbenzoate **vi** and **vii**.

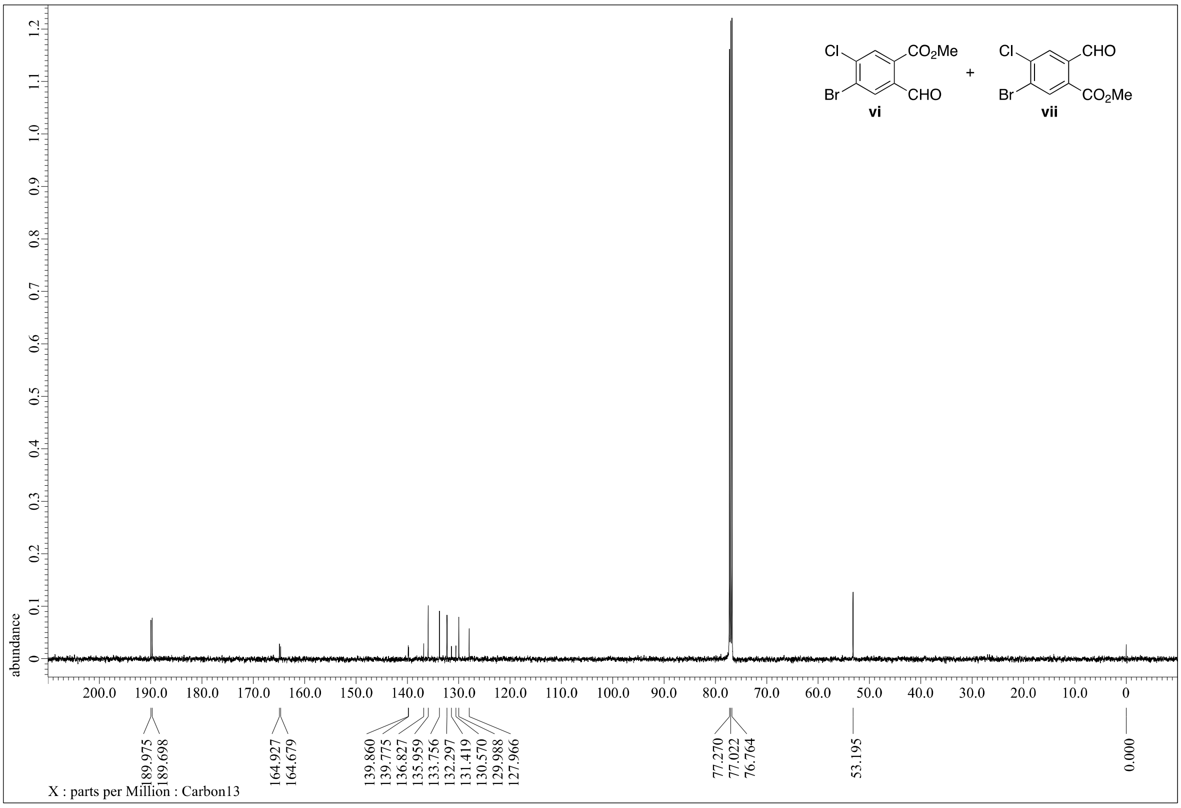

Figure S10. ^{13}C-NMR (500 MHz, $CDCl_3$) spectrum of methyl 2-formylbenzoate **vi** and **vii**.

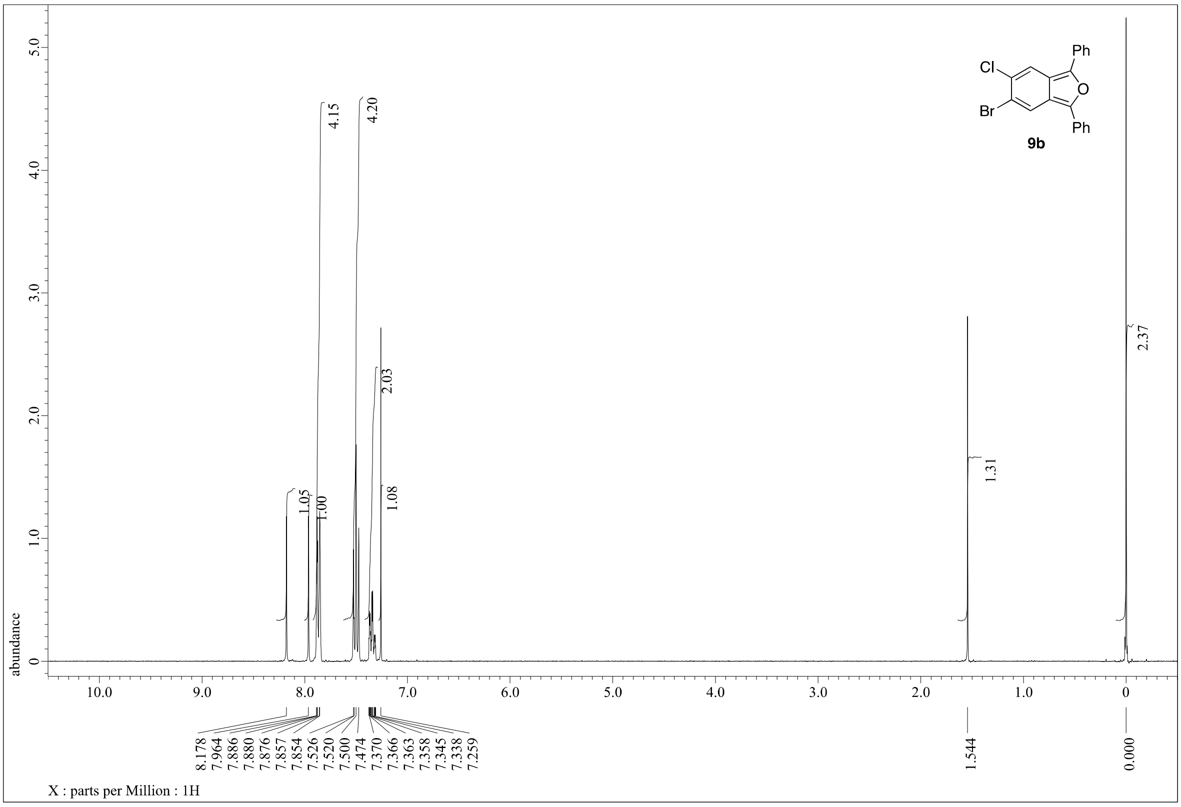

Figure S11. ^{1}H-NMR (300 MHz, $CDCl_3$) spectrum of isobenzofuran **9b**.

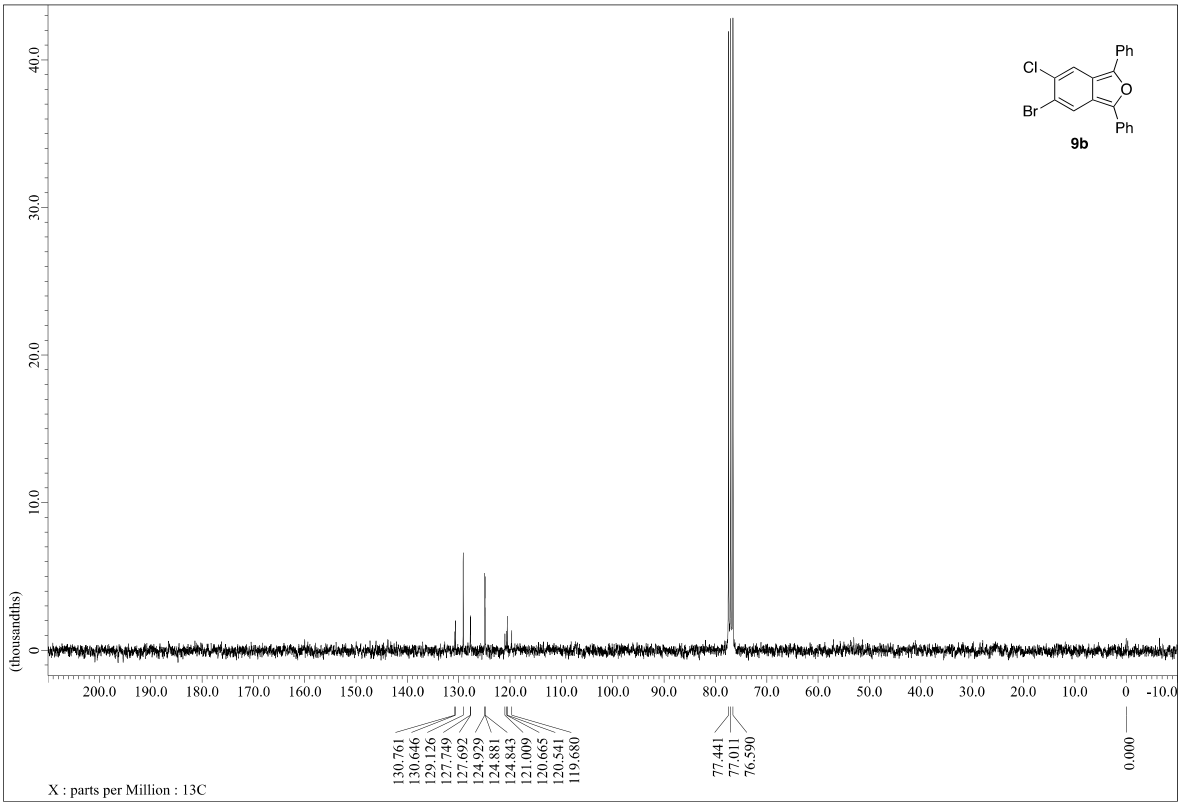

Figure S12. ^{13}C-NMR (300 MHz, $CDCl_3$) spectrum of isobenzofuran **9b**.

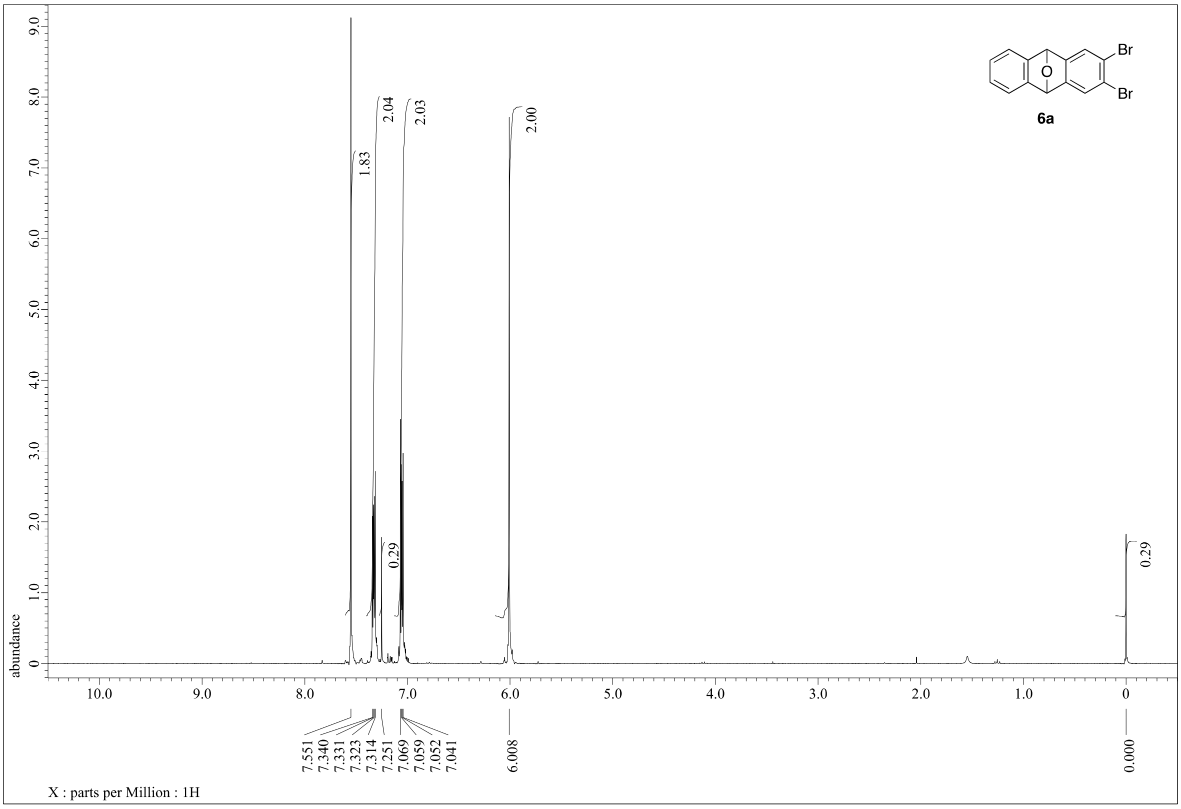

Figure S13. ^{1}H-NMR (300 MHz, $CDCl_3$) spectrum of cycloadduct **6a**.

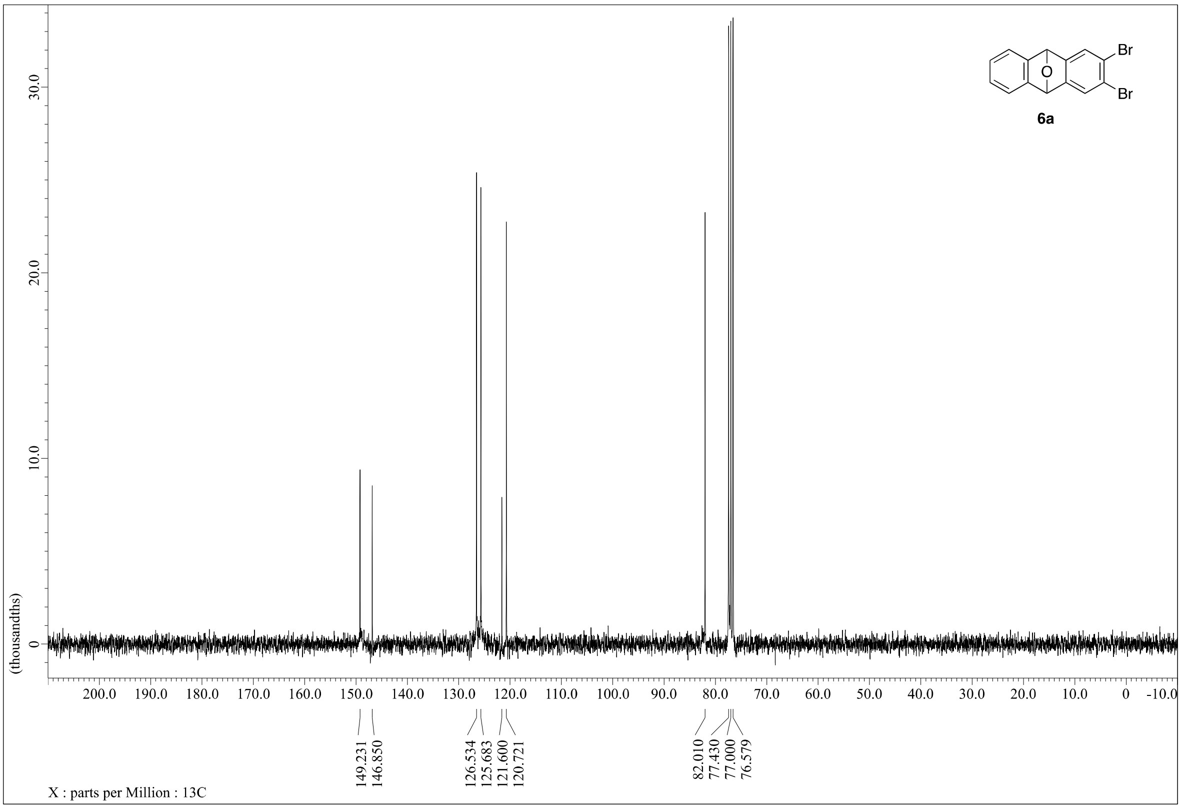

Figure S14. ^{13}C-NMR (300 MHz, $CDCl_3$) spectrum of cycloadduct **6a**.

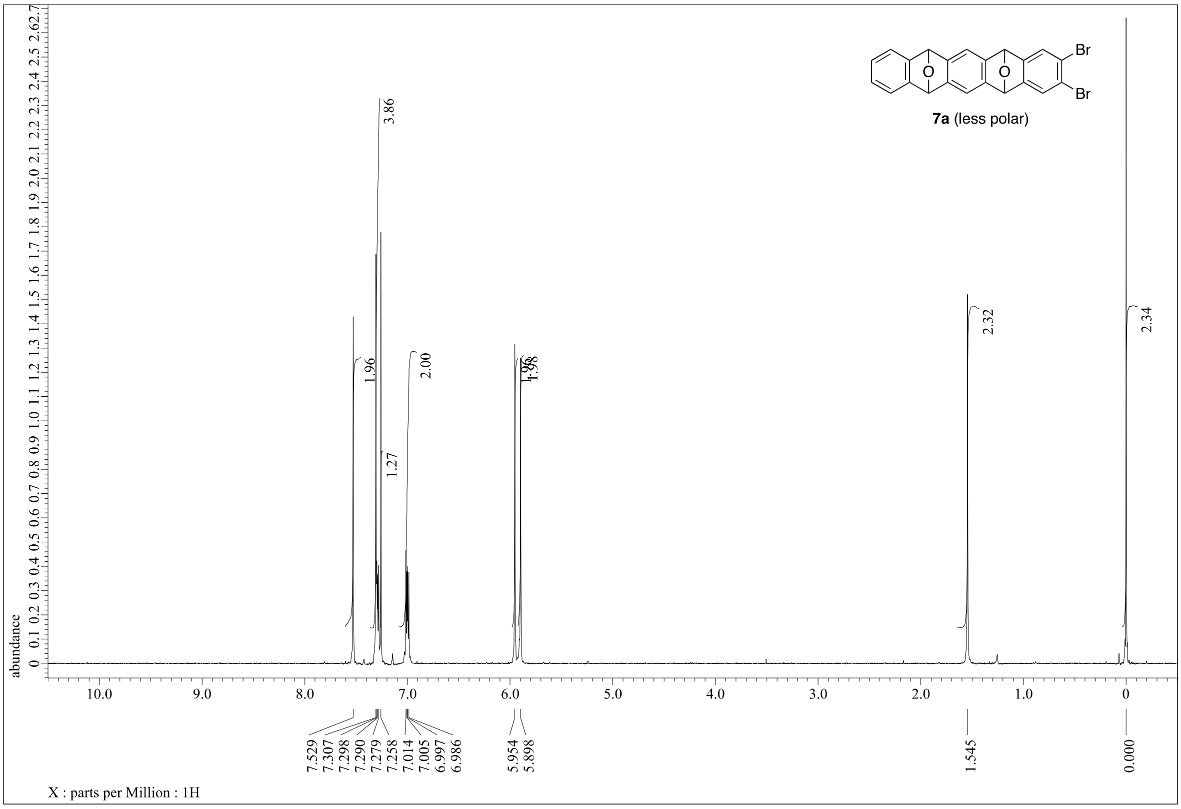

Figure S15. ^{1}H-NMR (300 MHz, $CDCl_3$) spectrum of cycloadduct **7a** (less polar).

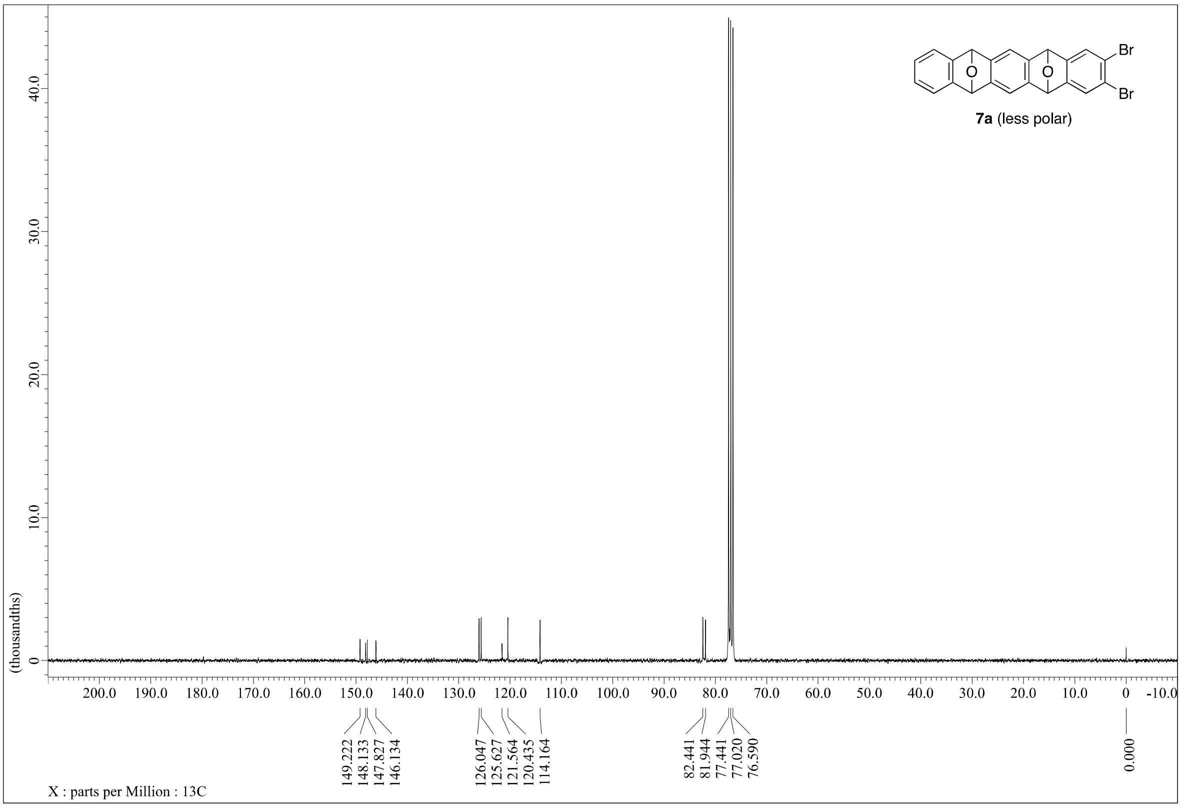

Figure S16. ^{13}C-NMR (300 MHz, $CDCl_3$) spectrum of cycloadduct **7a** (less polar).

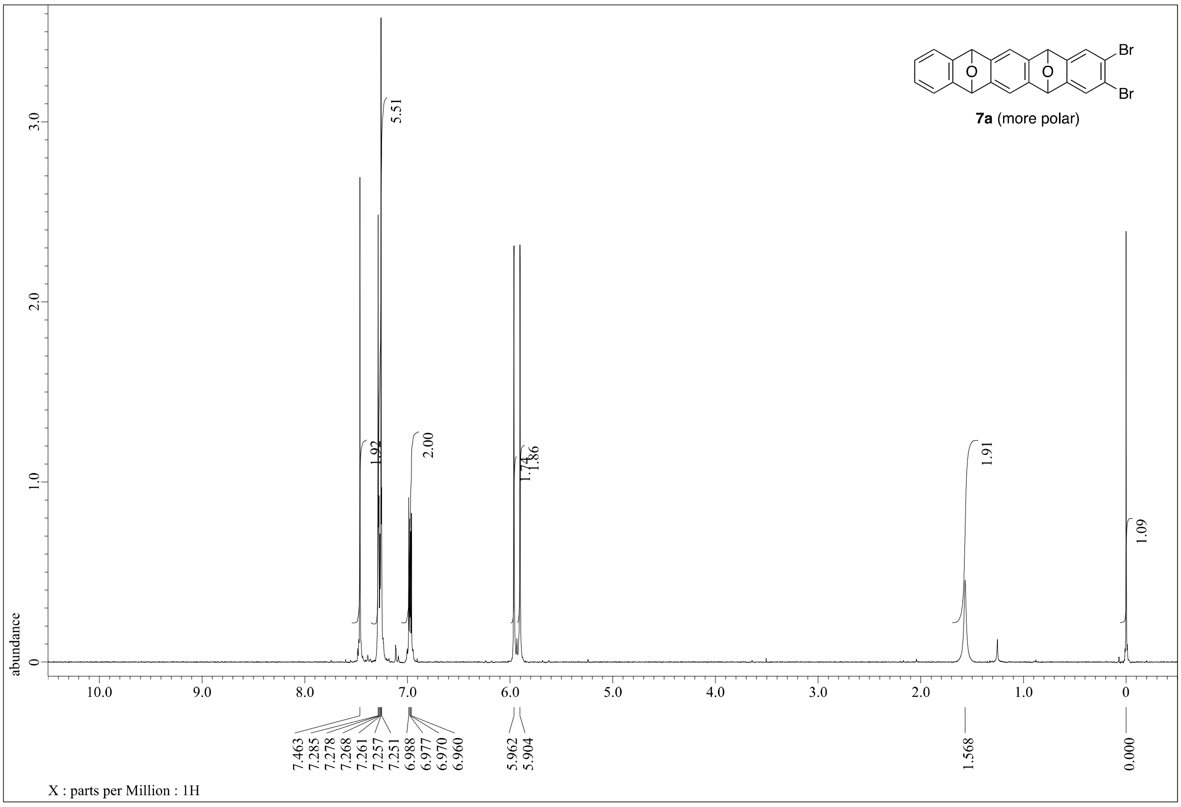

Figure S17. ^{1}H-NMR (300 MHz, $CDCl_3$) spectrum of cycloadduct **7a** (more polar).

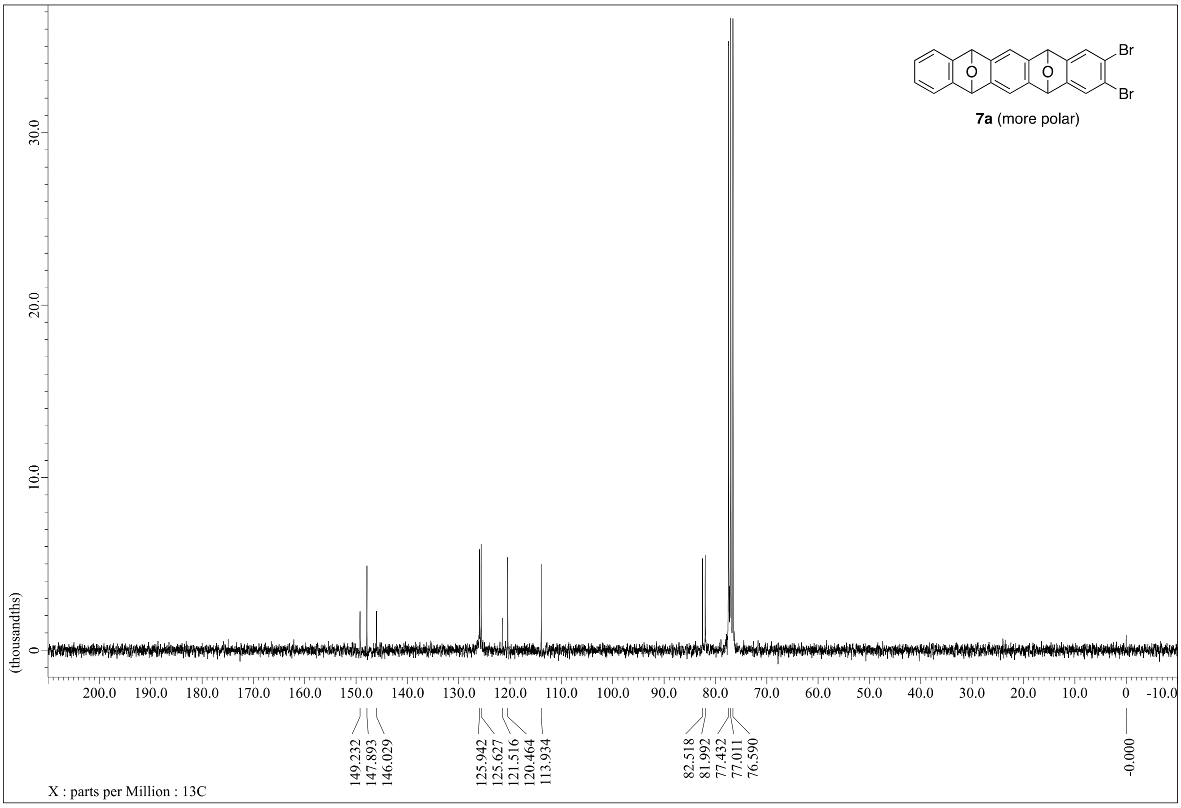

Figure S18. ^{13}C-NMR (300 MHz, $CDCl_3$) spectrum of cycloadduct **7a** (more polar).

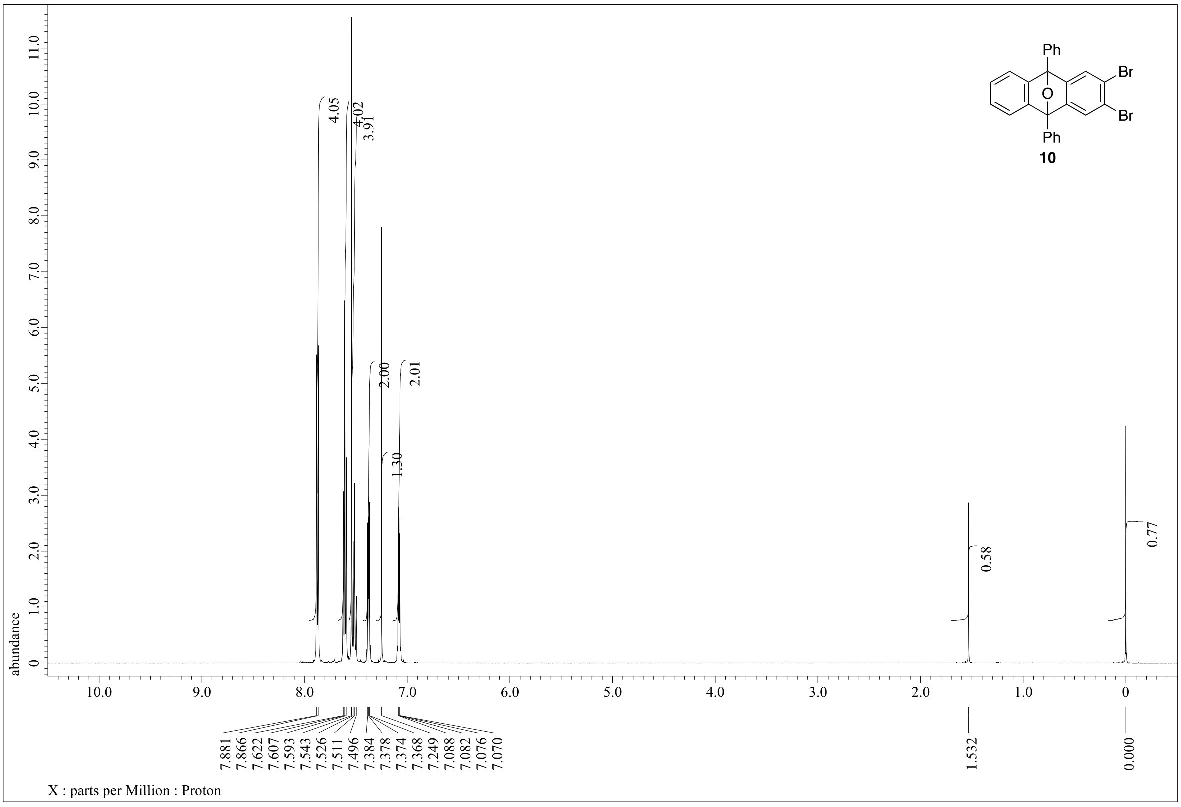

Figure S19. ^{1}H-NMR (500 MHz, $CDCl_3$) spectrum of cycloadduct **10**.

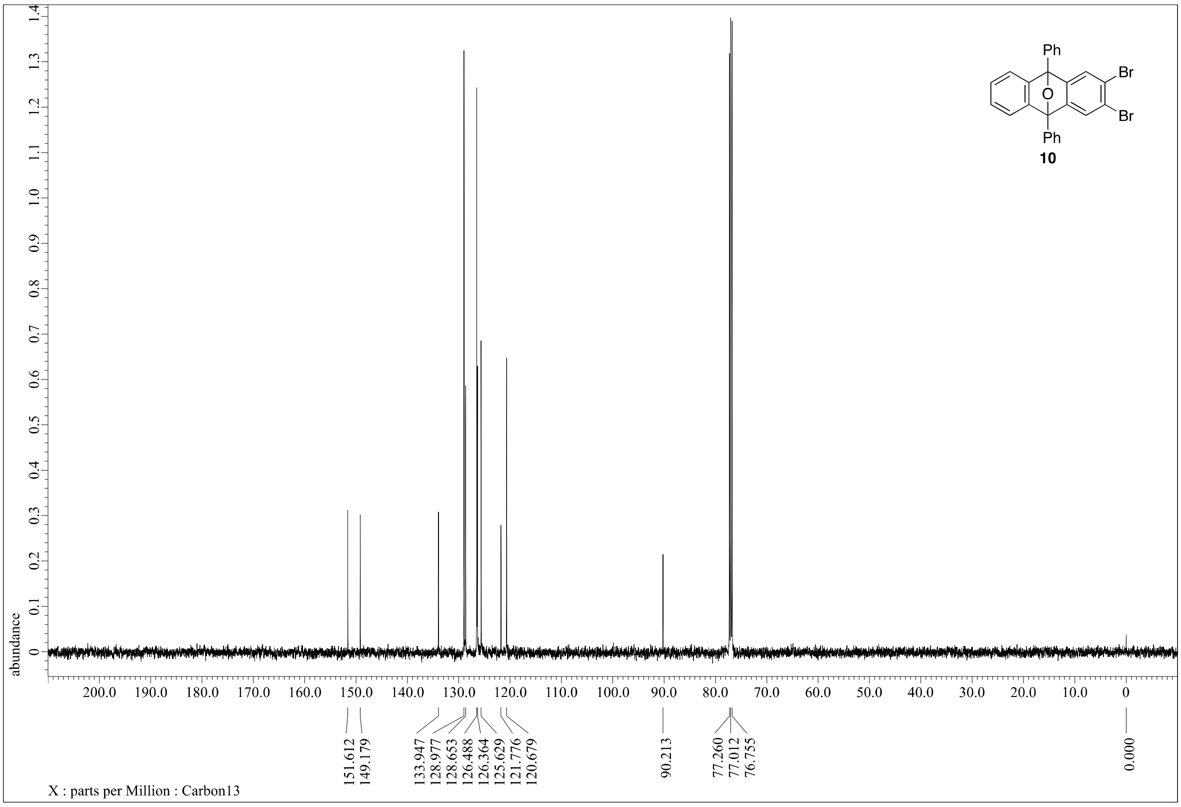

Figure S20. ^{13}C-NMR (500 MHz, $CDCl_3$) spectrum of cycloadduct **10**.

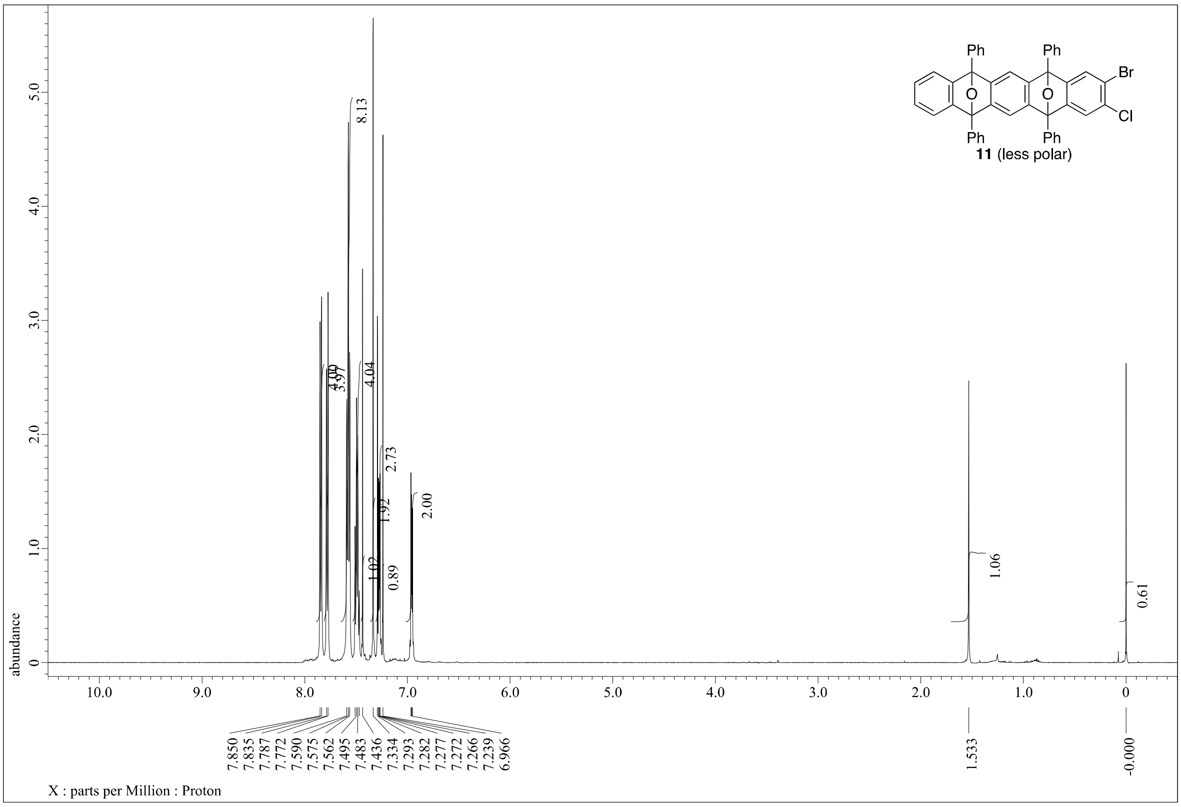

Figure S21. ^{1}H-NMR (500 MHz, $CDCl_3$) spectrum of cycloadduct **11** (less polar).

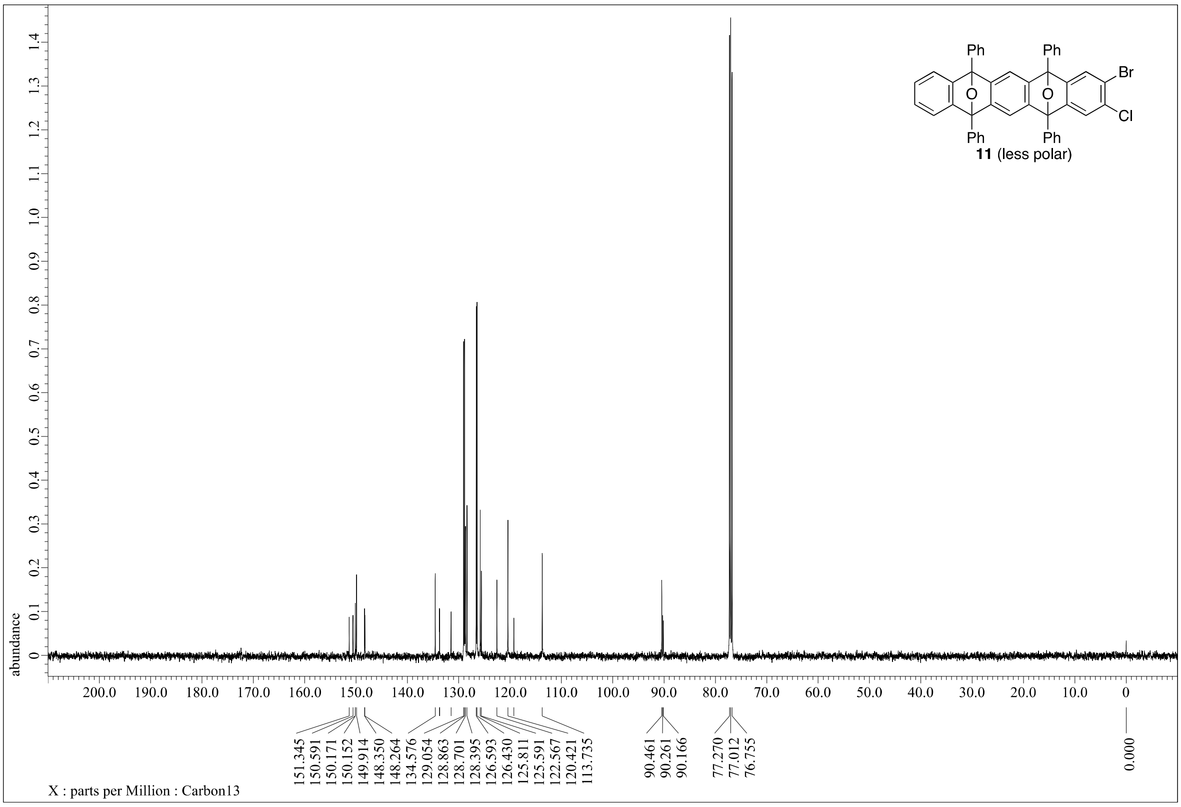

Figure S22. ^{13}C-NMR (500 MHz, $CDCl_3$) spectrum of cycloadduct **11** (less polar).

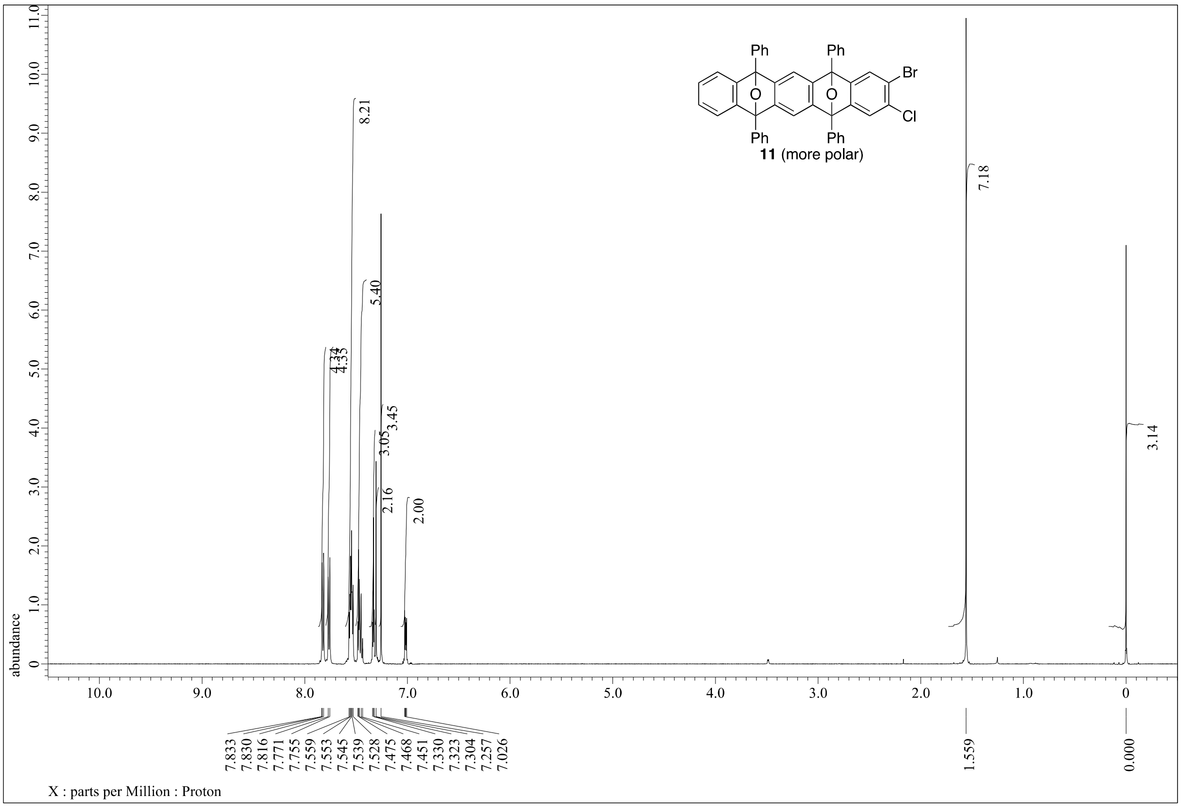

Figure S23. ^{1}H-NMR (500 MHz, $CDCl_3$) spectrum of cycloadduct **11** (more polar).

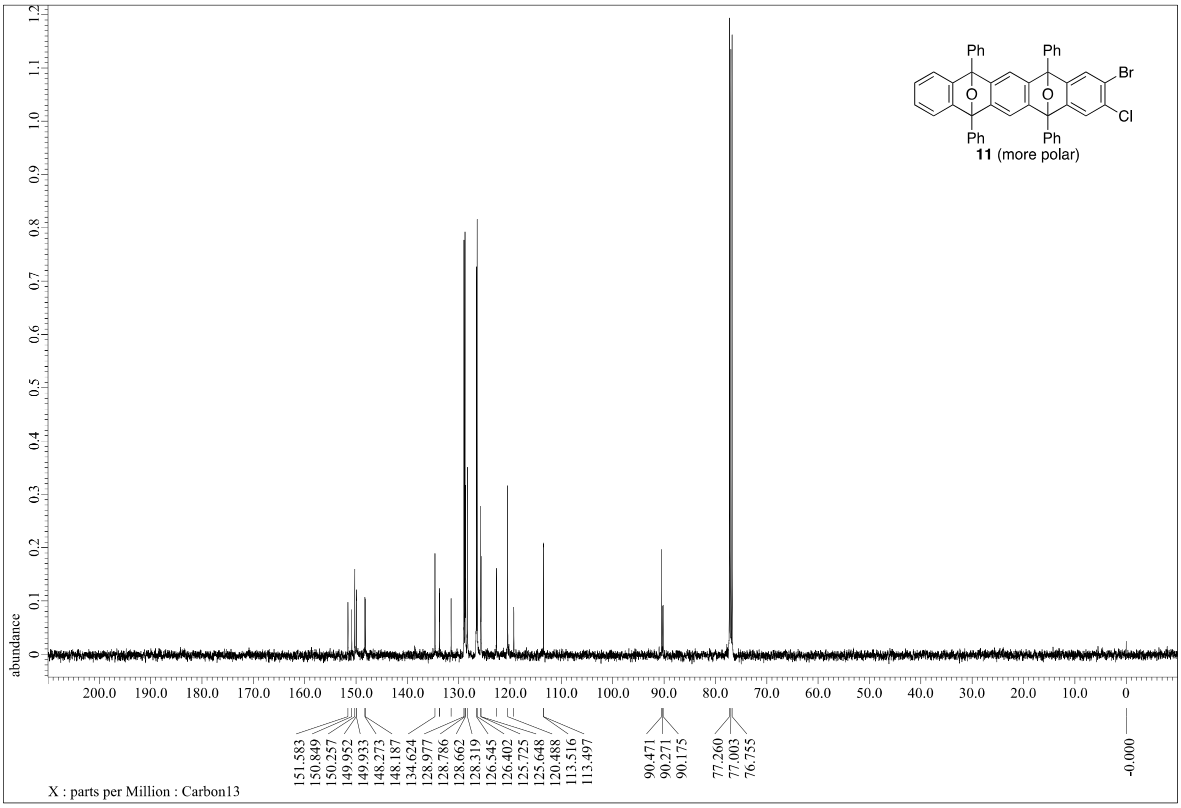

Figure S24. ^{13}C-NMR (500 MHz, $CDCl_3$) spectrum of cycloadduct **11** (more polar).

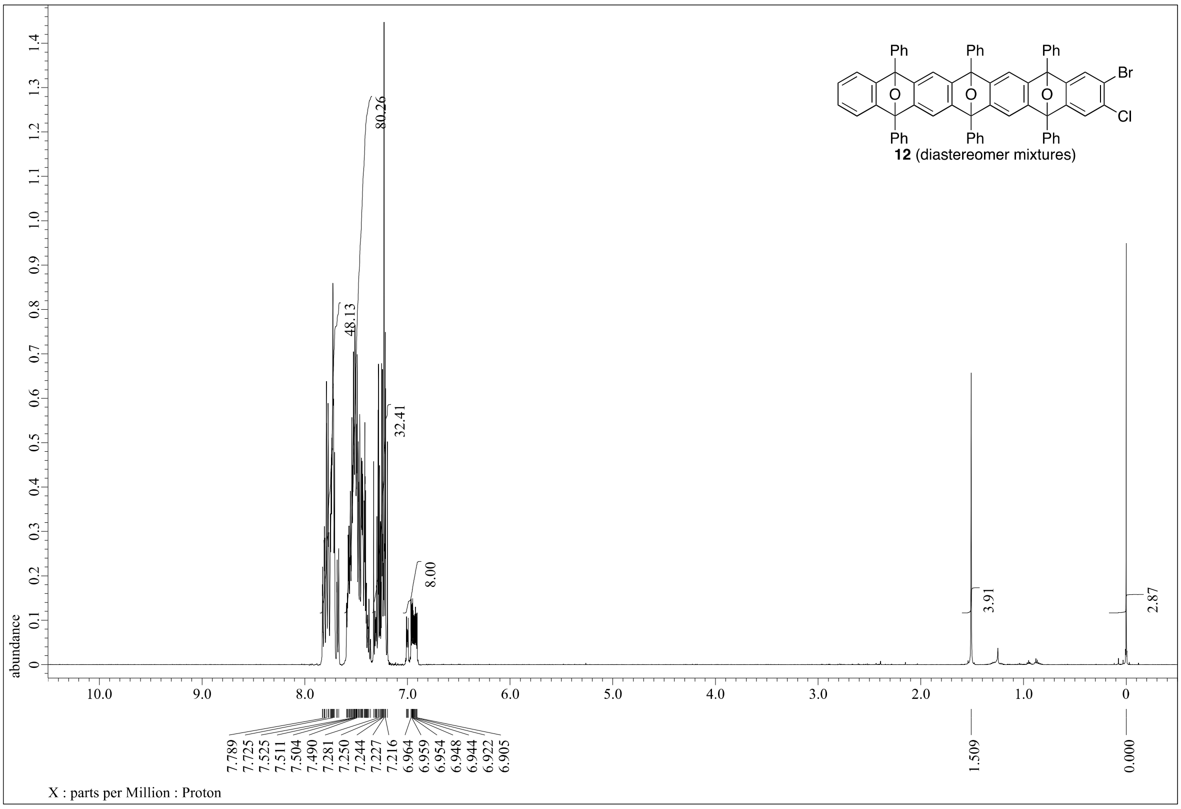

Figure S25. ^{1}H-NMR (500 MHz, $CDCl_3$) spectrum of cycloadduct **12** (a mixture of four diastereomers).

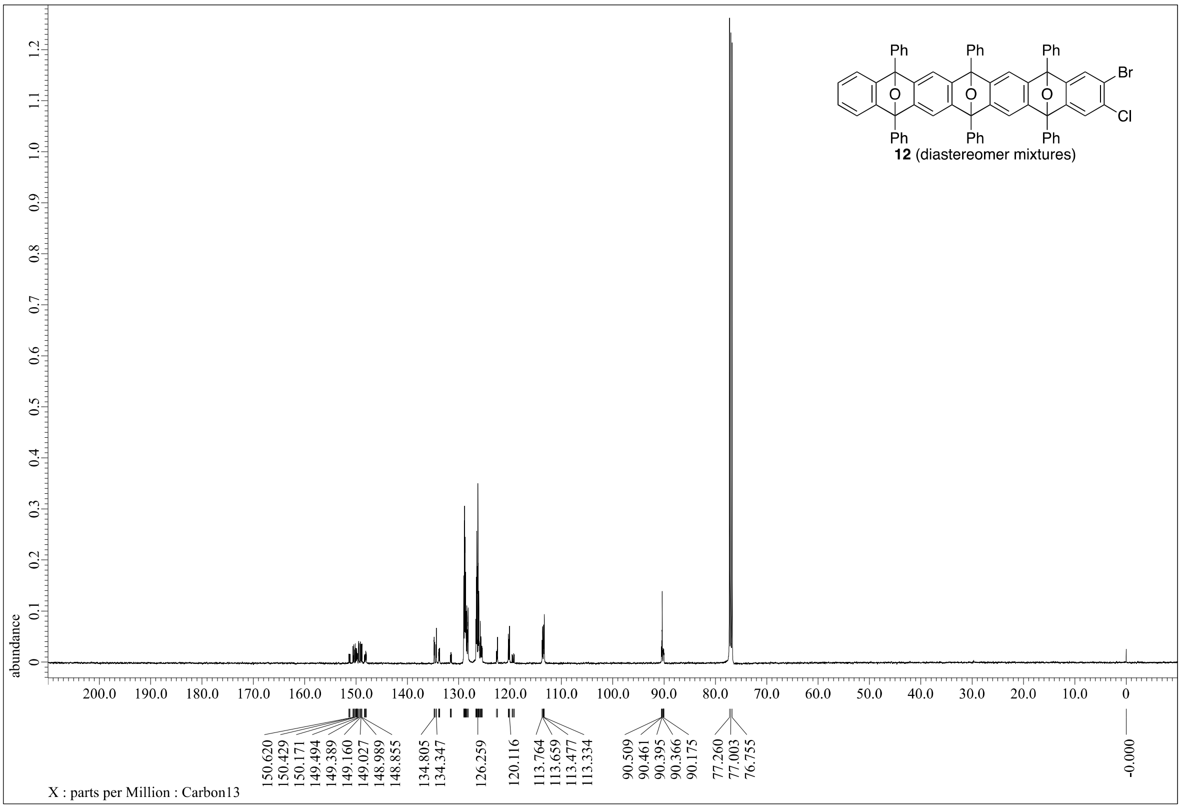

Figure S26. ^{13}C-NMR (500 MHz, $CDCl_3$) spectrum of cycloadduct **12** (a mixture of four diastereomers).

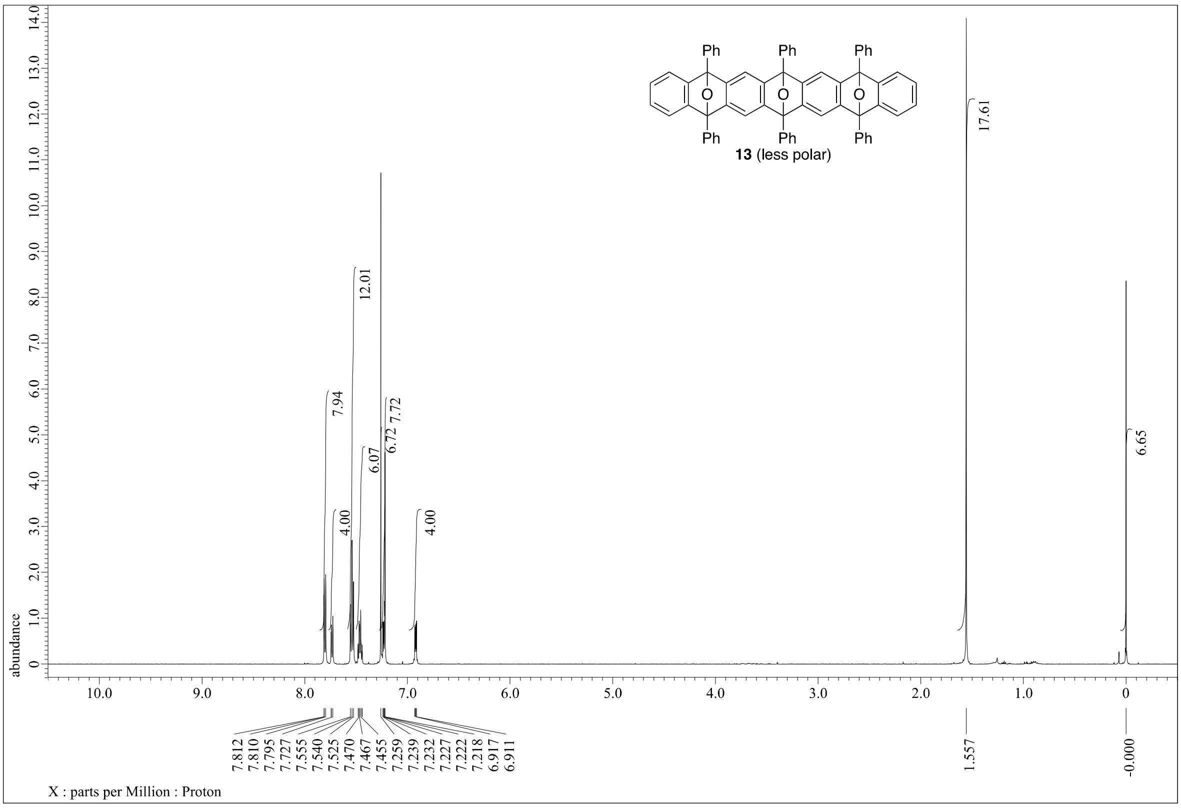

Figure S27. ^{1}H-NMR (500 MHz, $CDCl_3$) spectrum of cycloadduct **13** (less polar).

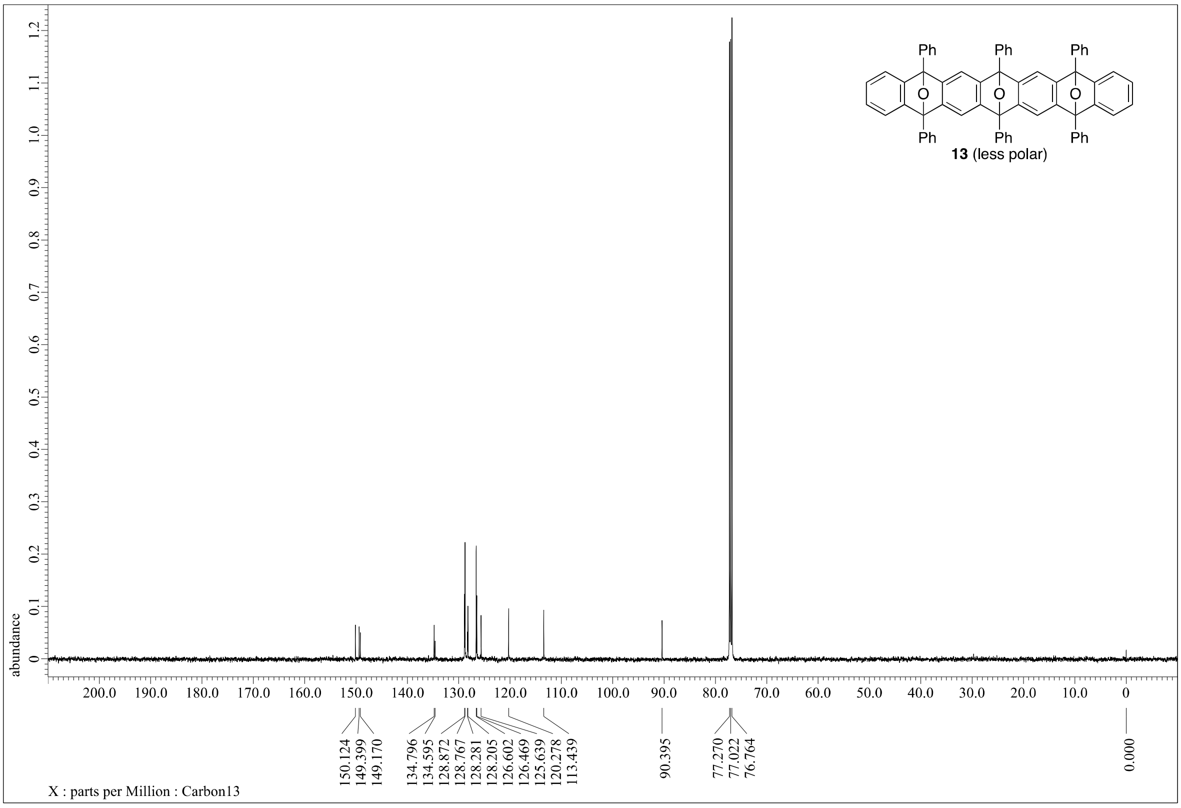

Figure S28. ^{13}C-NMR (500 MHz, $CDCl_3$) spectrum of cycloadduct **13** (less polar).

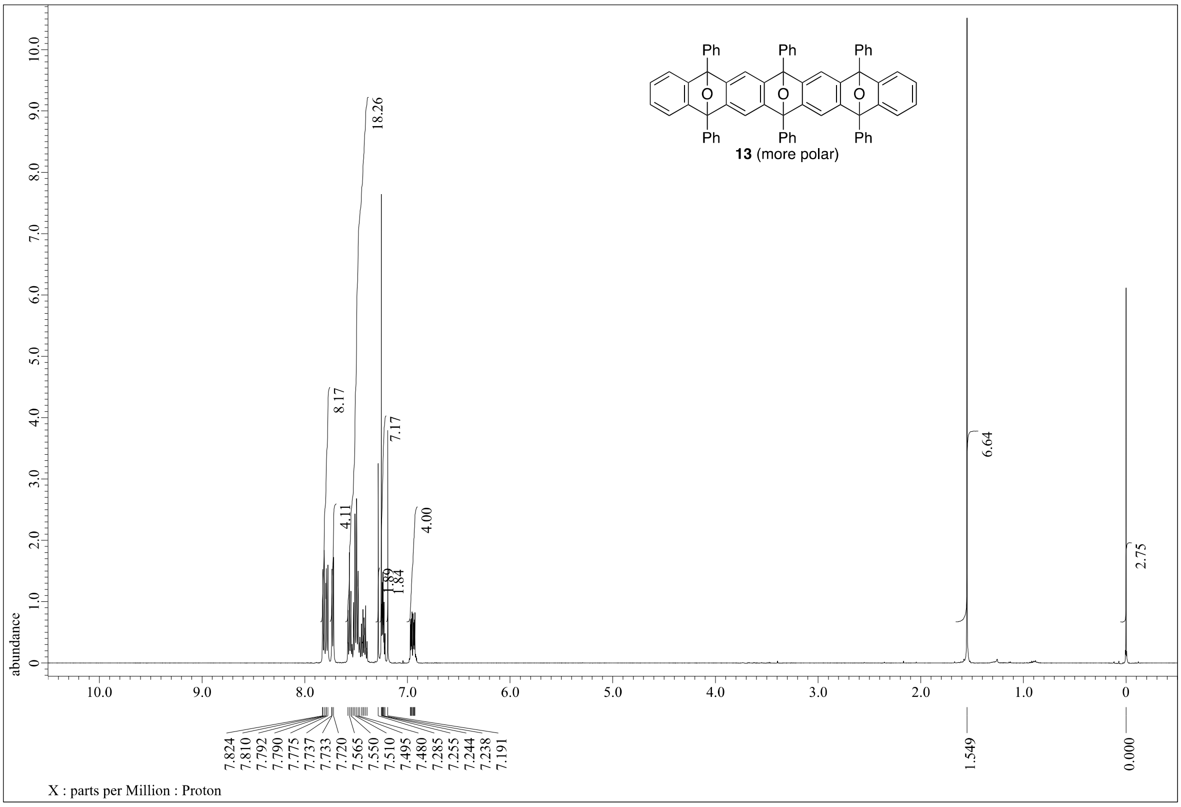

Figure S29. ^{1}H-NMR (500 MHz, $CDCl_3$) spectrum of cycloadduct **13** (more polar).

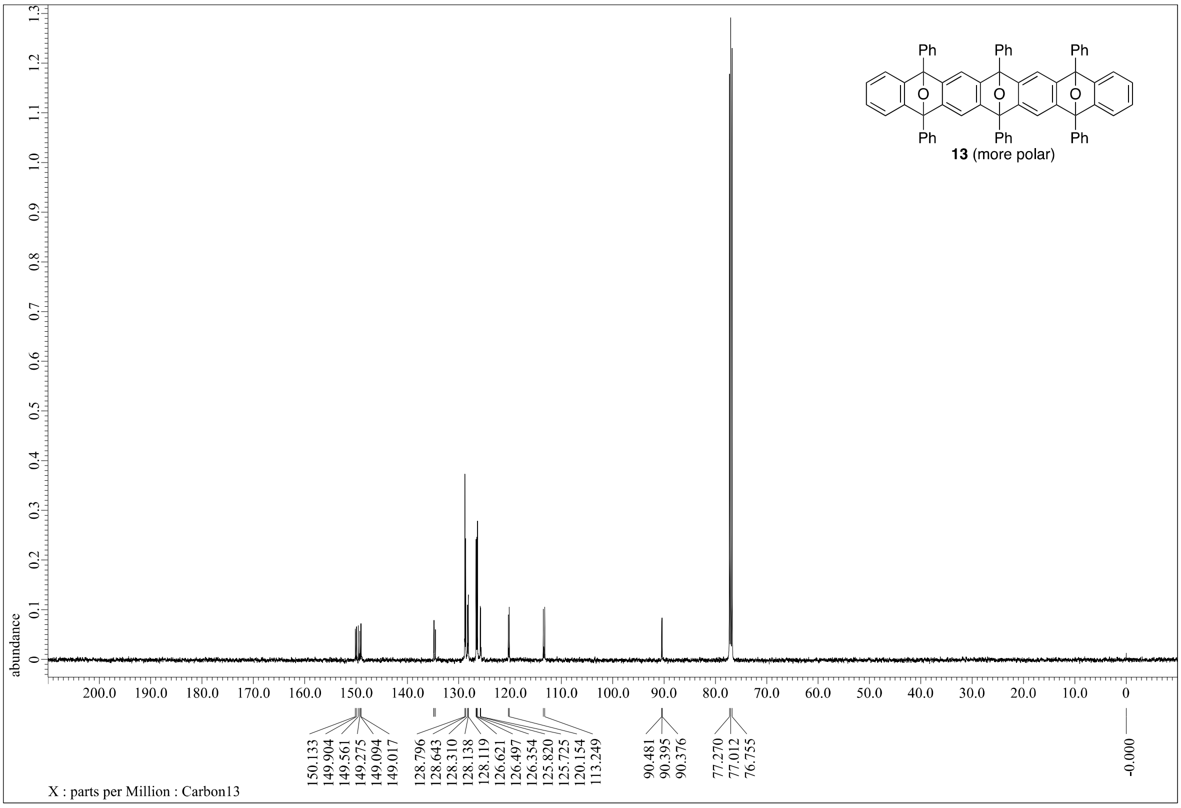

Figure S30. ^{13}C-NMR (500 MHz, $CDCl_3$) spectrum of cycloadduct **13** (more polar).

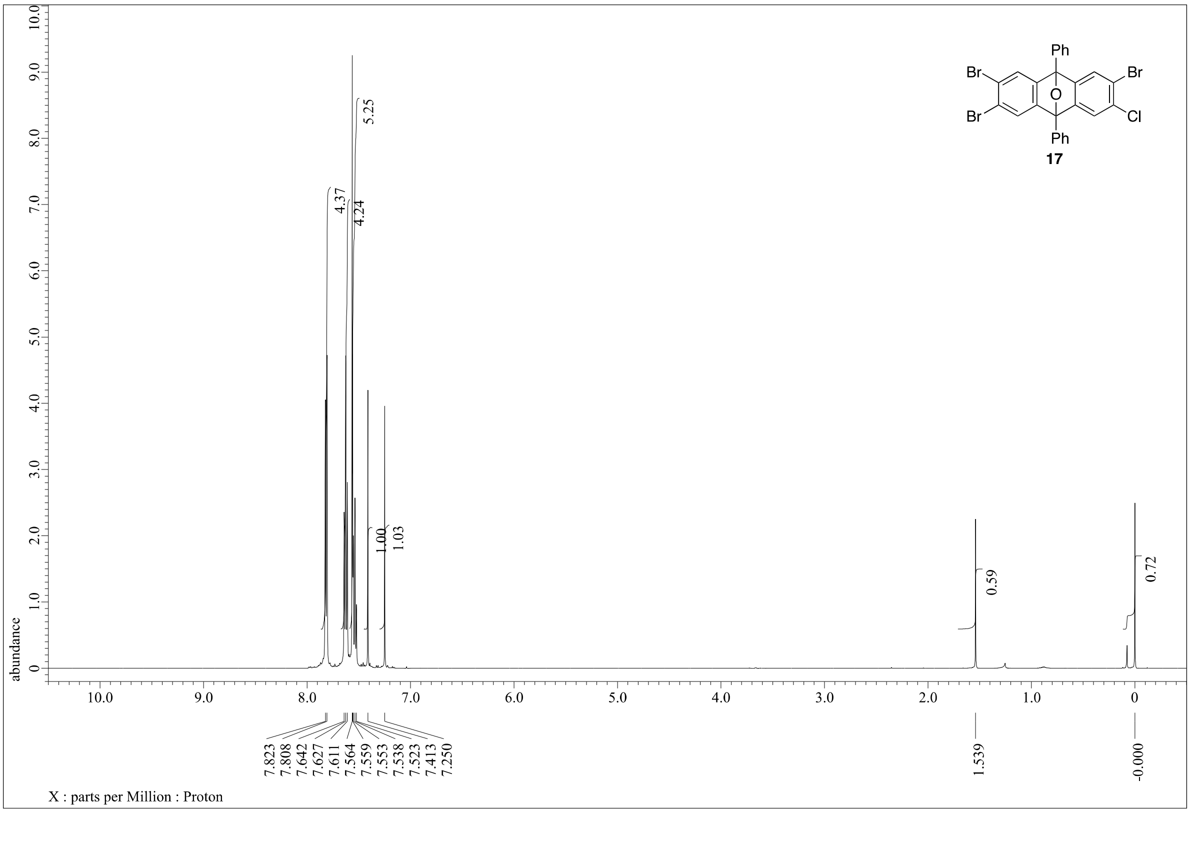

Figure S31. ^{1}H-NMR (500 MHz, $CDCl_3$) spectrum of cycloadduct **17**.

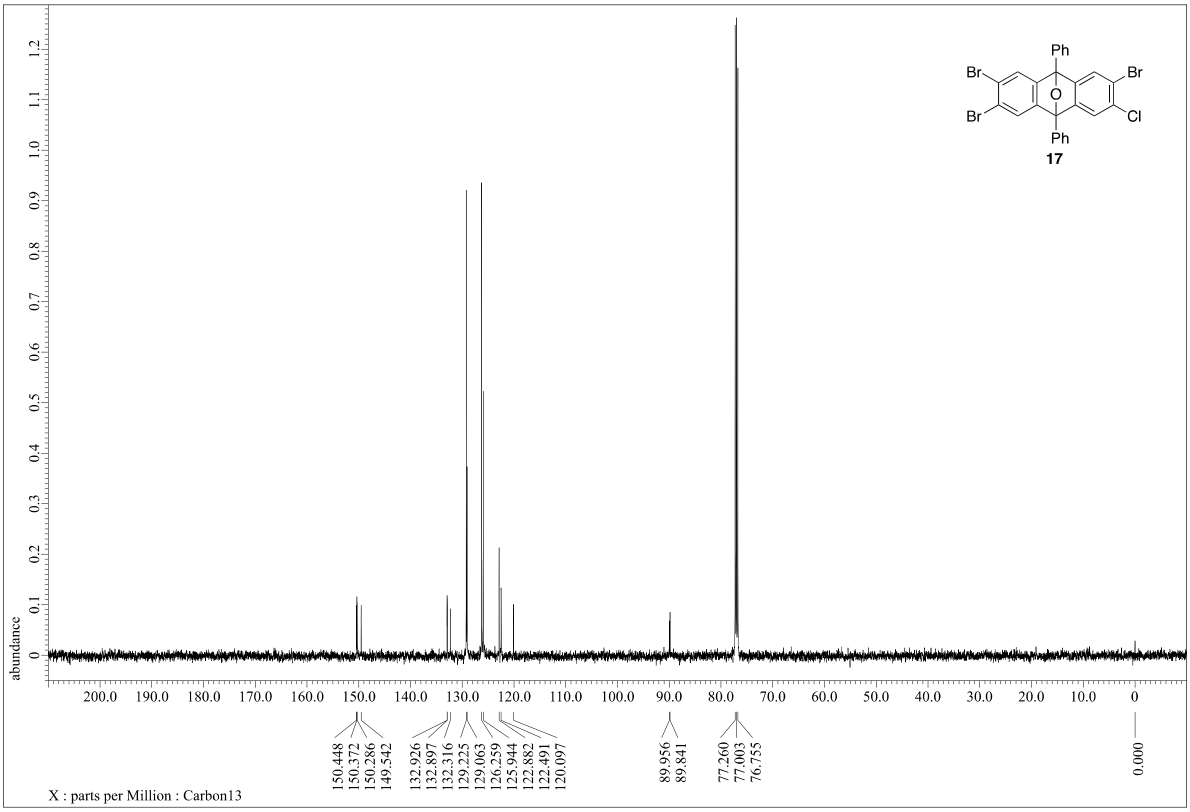

Figure S32. ^{13}C-NMR (500 MHz, $CDCl_3$) spectrum of cycloadduct **17**.

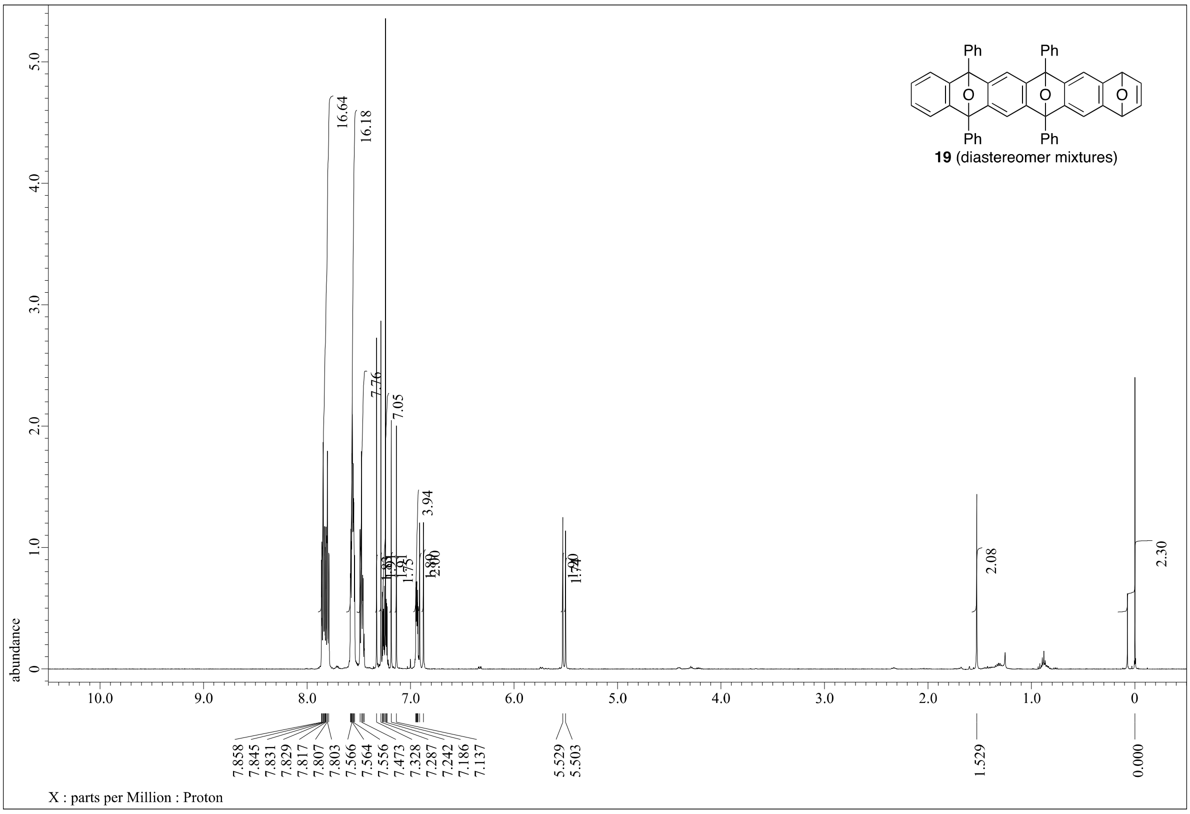

Figure S33. ^{1}H-NMR (500 MHz, $CDCl_3$) spectrum of cycloadduct **19** (a mixture of two diastereomers).

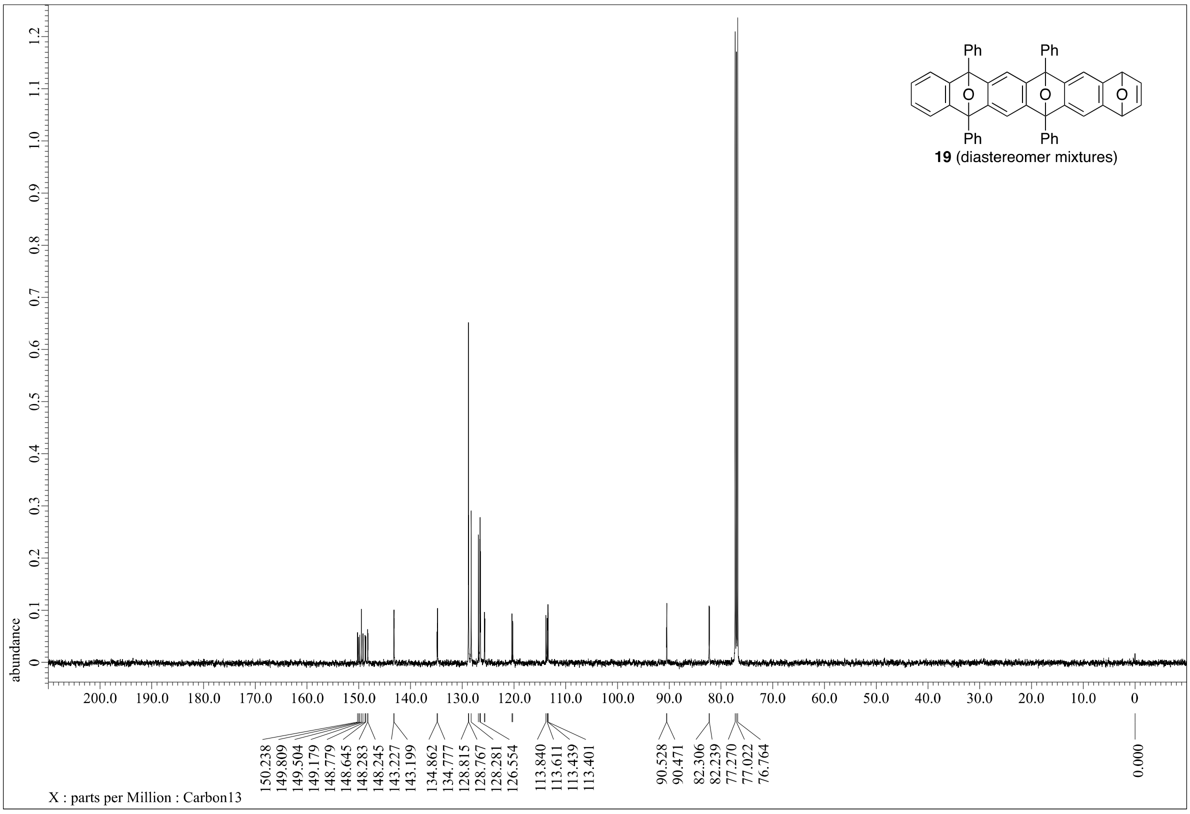

Figure S34. ^{13}C-NMR (500 MHz, $CDCl_3$) spectrum of cycloadduct **19** (a mixture of two diastereomers).

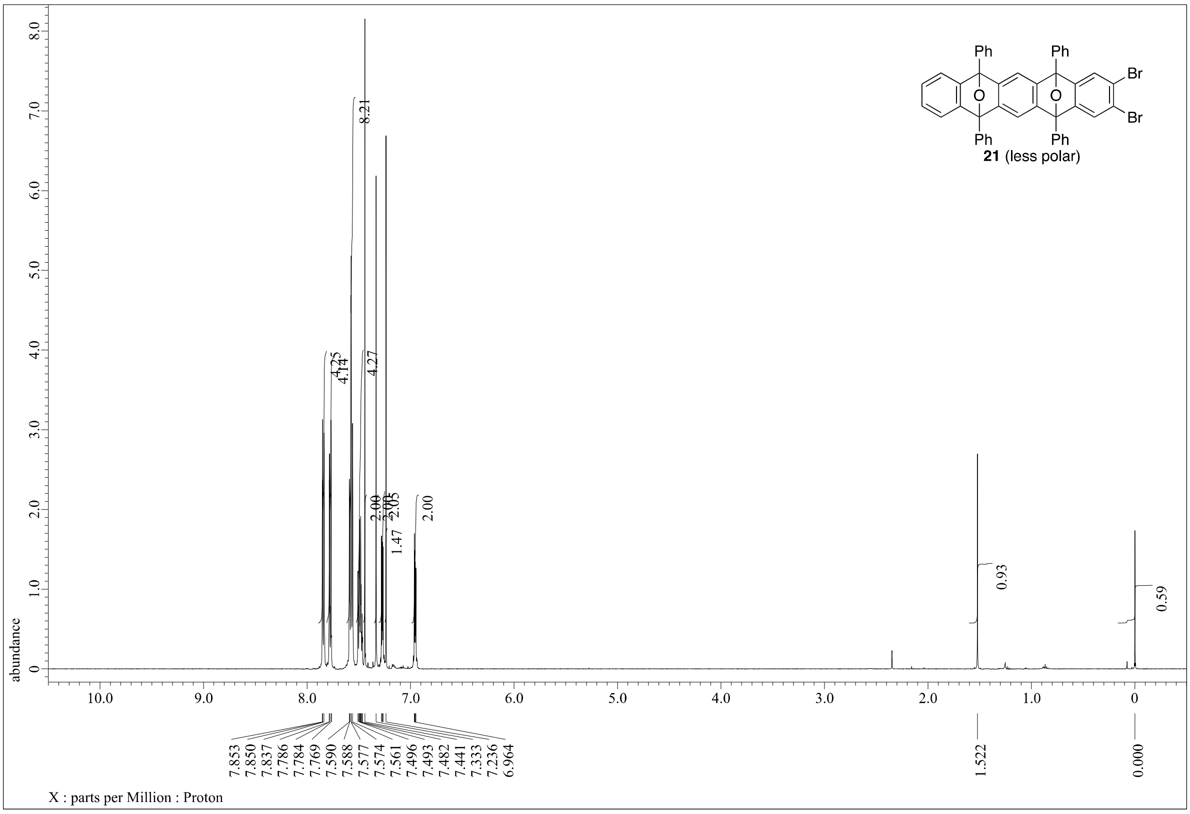

Figure S35. ^{1}H-NMR (500 MHz, $CDCl_3$) spectrum of cycloadduct **21** (less polar).

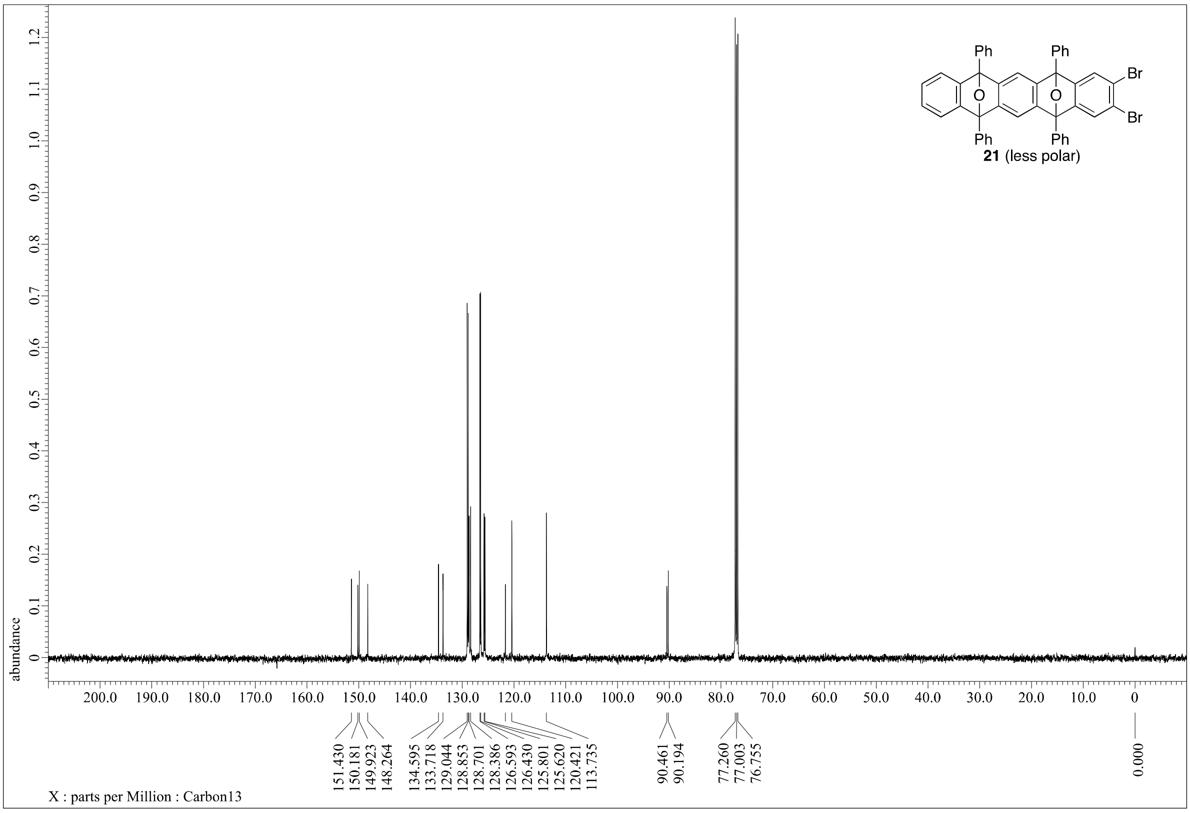

Figure S36. ^{13}C-NMR (500 MHz, $CDCl_3$) spectrum of cycloadduct **21** (less polar).

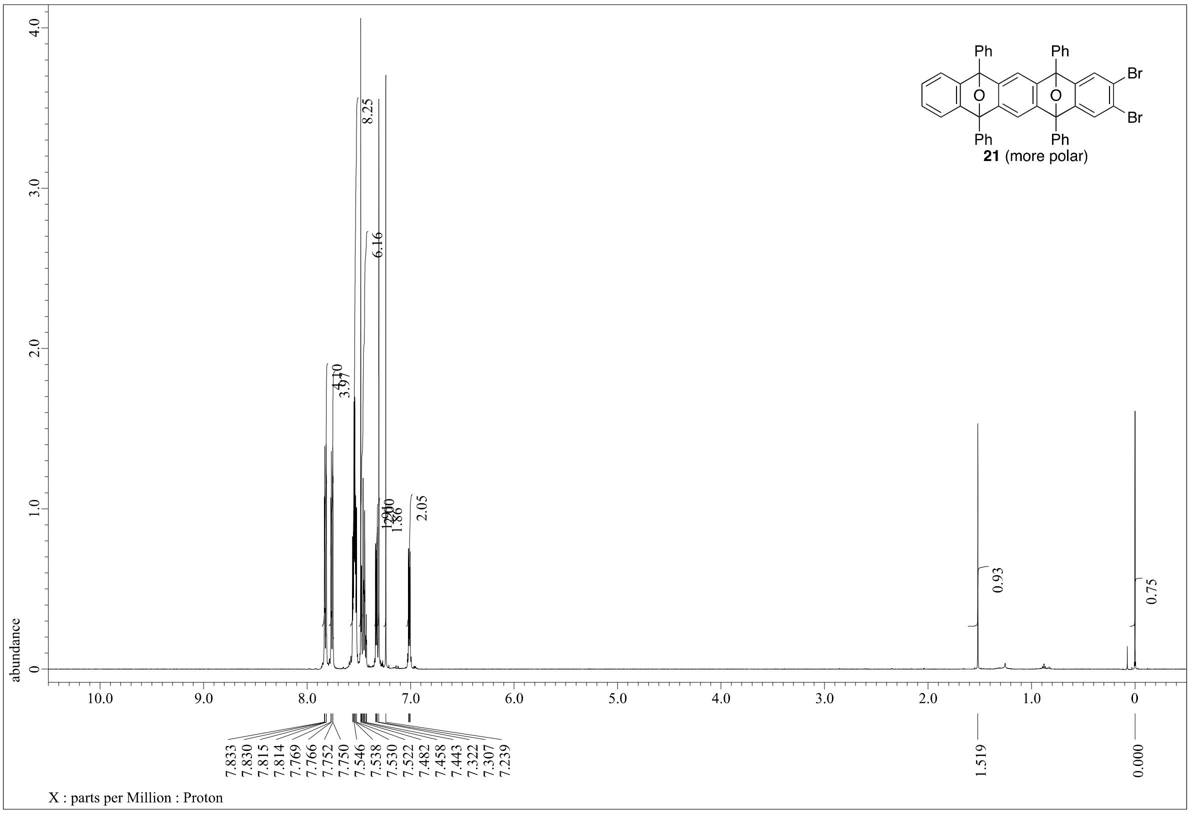

Figure S37. ^{1}H-NMR (500 MHz, $CDCl_3$) spectrum of cycloadduct **21** (more polar).

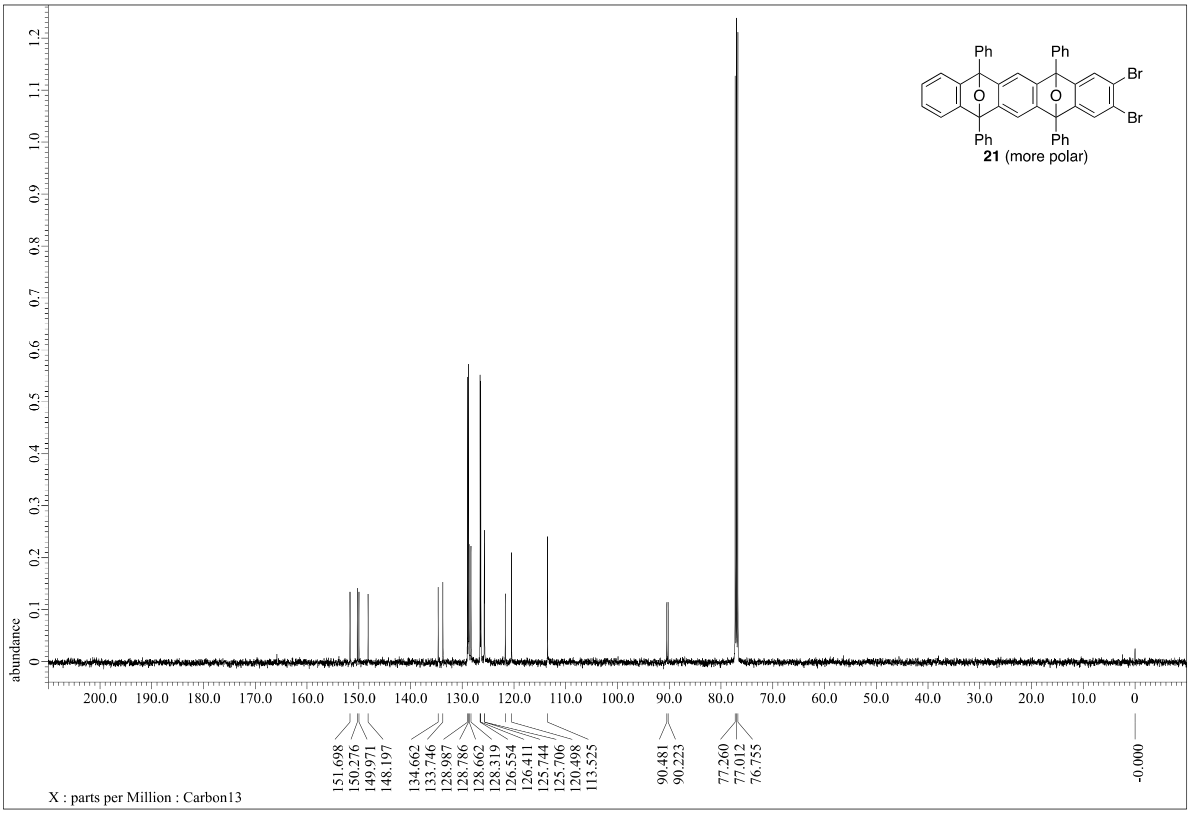

Figure S38. ^{13}C-NMR (500 MHz, $CDCl_3$) spectrum of cycloadduct **21** (more polar).
